# Sensory-Guided Establishment of Sensory Lexicon and Investigation of Key Flavor Components for Goji Berry Pulp

**DOI:** 10.3390/plants13020173

**Published:** 2024-01-08

**Authors:** Shuying Wang, Qingyu Su, Yuxuan Zhu, Jiani Liu, Xinke Zhang, Yu Zhang, Baoqing Zhu

**Affiliations:** 1 State Key Laboratory of Tree Genetics and Breeding, College of Biological Sciences and Technology, Beijing Forestry University, Beijing 100083, China; wsy15652658100@163.com; 2 Beijing Key Laboratory of Forestry Food Processing and Safety, Department of Food Science, College of Biological Sciences and Biotechnology, Beijing Forestry University, Beijing 100083, China; qingyusu1009@163.com (Q.S.); zhuyuxuan@bjfu.edu.cn (Y.Z.); liujn@bjfu.edu.cn (J.L.); 3 Food Science and Engineering College, Beijing University of Agriculture, Beijing 102206, China; zhangxinke@bua.edu.cn; 4 “The Belt and Road” International Institute of Grape and Wine Industry Innovation, Beijing University of Agriculture, Beijing 102206, China

**Keywords:** goji berry pulp, sensory lexicon, aroma-active compounds, HS-SPME-GC-Orbitrap-MS, PLSR

## Abstract

Many customers prefer goji berry pulp, well-known for its high nutritional content, over fresh goji berries. However, there is limited research on its sensory lexicon and distinctive flavor compounds. This study focused on developing a sensory lexicon for goji berry pulp and characterizing its aroma by sensory and instrumental analysis. Sensory characteristics of goji berry pulp were evaluated by our established lexicon. A total of 83 aromatic compounds in goji berry pulp were quantified using HS-SPME-GC-Orbitrap-MS. By employing OAV in combination, we identified 17 aroma-active compounds as the key ingredients in goji berry pulp. Then, we identified the potentially significant contributors to the aroma of goji berry pulp by combining principal component analysis and partial least squares regression (PLSR) models of aroma compounds and sensory attributes, which included 3-ethylphenol, methyl caprylate, 2-hydroxy-4-methyl ethyl valerate, benzeneacetic acid, ethyl ester, hexanal, *(E,Z)*-2,6-nonadienal, acetylpyrazine, butyric acid, 2-ethylhexanoic acid, 2-methyl-1-propanol, 1-pentanol, phenylethyl alcohol, and 2-nonanone. This study provides a theoretical basis for improving the quality control and processing technology of goji berry pulp.

## 1. Introduction

Goji berry (*Lycium barbarum* L.), a deciduous shrub, is widely distributed in arid and semi-arid regions of Northwestern China, Southeastern Europe, and the Mediterranean region [1,2]. The berries are abundant in goji polysaccharides, carotenoids, organic acids, anthocyanins, flavonoids, phenolic acids, and fatty acids [3,4,5,6,7]. They are a healthy food item with medicinal and nutritional values and a traditional Chinese herbal medicine.

The scale of cultivation is expanding in tandem with the increasing demand for consumption. Goji berries are easily damaged or spoiled during storage and transportation due to their soft tissue, which is highly vulnerable to mechanical damage and microbial infection. Goji berries are processed into various products, including juice, wine, tea, concentrated tablets, etc. [8,9]. Due to its ability to retain nutritional value, goji berry pulp has recently become a popular new product in the food industry. Goji berry pulp is the fresh cloudy juice extracted from goji berries without the addition of any preservatives or chemicals. It is produced through a series of steps, including selecting, cleaning, crushing, pulping, filtering, and homogenizing the fresh goji berries [10].

Nowadays, Goji berry pulp has become increasingly popular among customers due to its superior nutritional value. In addition, flavor is also one of the most essential elements in luring customers and enhancing food quality [11]. The sensory evaluation aims to analyze the qualities of a product by carefully weighing all of its perceivable characteristics and their relative intensities. It is commonly used in product development, quality control, optimization, and grading. A sensory lexicon is a vocabulary list that rationally defines a food’s sensory characteristics, and is created using standardized vocabulary [12]. The need for sensory lexicons originates from multiple stages of the food industry, such as research and development, production, quality control, product shelf life, and marketing [13]. It makes it possible for various people to describe food using a lexicon that follows standardized procedures [14]. Many fruits and juices, such as blueberry and raspberry [13], *Actinidia arguta* fruits [15], strawberry [16], pomegranate [17], and blueberry [18], use sensory lexicons extensively. A specialized sensory lexicon for goji berry pulp is urgently needed because it is necessary to build a standardized sensory evaluation system for goji berry pulp products.

The aroma quality is a crucial factor that influences the flavor characteristics and quality of goji berries and their products, which significantly affects consumer preference and acceptance of the products [19]. Although Chen et al. [20], Yuan et al. [21], and Liu et al. [22] analyzed volatile aroma compounds in goji berry fruit samples, yeast-fermented fresh goji berry juice samples, and lactobacillus-fermented goji berry juice samples, no relevant research has been performed on goji berry pulp products. In addition, previous studies frequently identified the volatile aroma compounds in juice using the headspace solid-phase microextraction and gas chromatography-mass spectrometry (GC-MS) technology. However, the limited resolution and sensitivity of the technique restricted a comprehensive analysis of the volatile profile. GC-MS with high-resolution accurate mass orbitrap detection (GC-Orbitrap-MS) is a new GC-MS technique that can provide high-quality resolution (120,000 full width at half maximum (m/z 200) and high-quality accuracy (<3 ppm)), and is a highly sensitive analytical method. The GC-Orbitrap-MS-based quantitative analysis of pesticide residues has been applied extensively in food products such as fruits, vegetables, and grains [23]. Then, Liu et al. [24] developed a suitable method for GC-Orbitrap-MS to determine trace compounds in fruit wines. They discovered this method was effective for quantitative analysis and targeted screening because it could identify a wide range of lactones and volatile phenolic compounds in fruit wines, including goji berry wine. To the best of our knowledge, no studies have used GC-Orbitrap-MS to analyze aroma compounds in goji berry pulp.

Moreover, there is still a lack of comprehensive studies on the aroma composition and sensory characteristics of goji berry pulp at the present time. Therefore, a specialized lexicon for goji berry pule was first established by a professional sensory panel, which included aroma, flavor, and mouthfeel attributes. Then, the volatile aroma compounds in goji berry pulp were identified using the HS-SPME-GC-Orbitrap-MS method, and the odor activity values (OAV) values were calculated to analyze how each volatile aroma compound contributed to the aroma characteristics of the samples. Finally, a PLSR model was established to investigate the relationship between volatile aroma compounds and aroma attributes, so as to analyze the material basis of the different aroma attributes of goji berry pulp.

## 2. Results and Discussion

### 2.1. pH, Soluble Solids, Organic Acids, and Reducing Sugars

Table 1 displays the pH and soluble solid contents of seven goji berry pulp samples. The soluble solids ranged from 12.83 to 20.70, while the pH ranged between 4.00 and 4.41. Fruit juice sourness was primarily attributed to organic acids [25], of which goji berry pulp samples contained five kinds. The various goji berry pulp samples had varying levels of oxalic acid (0.10–0.73 g/L), tartaric acid (1.40–2.16 g/L), malic acid (1.80–5.20 g/L), lactic acid (1.06–3.34 g/L), and citric acid (3.14–8.37 g/L). Citric and malic acids were highest in the samples compared to other organic acids. This is consistent with the findings of Oğuz et al. [26], where several goji berry genotypes displayed the highest levels of citric and malic acids. The sugar content in goji berry pulp, as indicated by the results of this study, ranged from 50.04 to 96.60 g/L for fructose and from 28.00 to 53.70 g/L for glucose. Although there have been no specific reports on the sugar content in goji berry pulp, previous studies showed that sugars occupied more than 50% of goji berries, contributing to the specific taste and the predominant nutritional components of goji berries [27]. Similarly, Zhao et al. [28] discovered that glucose and fructose were the major sugars present in goji berries. Furthermore, researchers also found that fructose and glucose could serve as the primary biomarkers for distinguishing goji berries from different geographical origins [27].

Overall, the analysis of pH, soluble solids, organic acids, and sugars in the goji berry pulp samples provides valuable information about their composition and can contribute to a better understanding of the flavor and quality characteristics of these fruits.

### 2.2. Establishment of Goji Berry Pulp Sensory Lexicon

A sensory lexicon is used for new product development, quality control, and product improvement, and greatly facilitates communication between people with different roles, such as sensory scientists, product developers, and technicians [29,30,31]. All descriptors were gathered and combined to create 55 descriptors for the open-ended questions test, including 25 descriptors related to aroma and 30 descriptors related to flavor/mouthfeel. After assessing the strength of each sensory descriptor, the panel calculated its M-value and eliminated those with M-values less than 0.200 (Table 2). Following the screening process, a total of 17 sensory descriptors were retained and included 9 aroma descriptors such as “goji berry” (0.609, M-values), “tomato” (0.571), “roast sweet potatoes” (0.514), “pumpkin” (0.397), “hay” (0.254), “sweet” (0.634), “acetic acid” (0.207), “honey” (0.406), and “jujube” (0.246) and 8 flavor/mouthfeel descriptors, such as “acidity” (0.555), “sweetness” (0.558), “bitter” (0.339), “astringency” (0.283), “goji berry” (0.471), “tomato” (0.394), “granularity” (0.430), and “viscosity” (0.680).

Afterward, we gave each sensory attribute a sensory reference once we had a consensus to help panelists during the sensory evaluation process. Many academics have studied the use of references in descriptive analysis in great detail, looking at the benefits and drawbacks of doing so [32]. In the present study, citing sources was essential to understand the attributes perceived here clearly. To do this, we used both natural references and chemical compounds. Through the above process, the sensory lexicon for aroma and flavor/mouthfeel is established as shown in Table 3.

### 2.3. Sensory Analysis

#### 2.3.1. Principal Component Analysis (PCA) of Sensory Characteristics of Goji Berry Pulp

**Figure 1:**
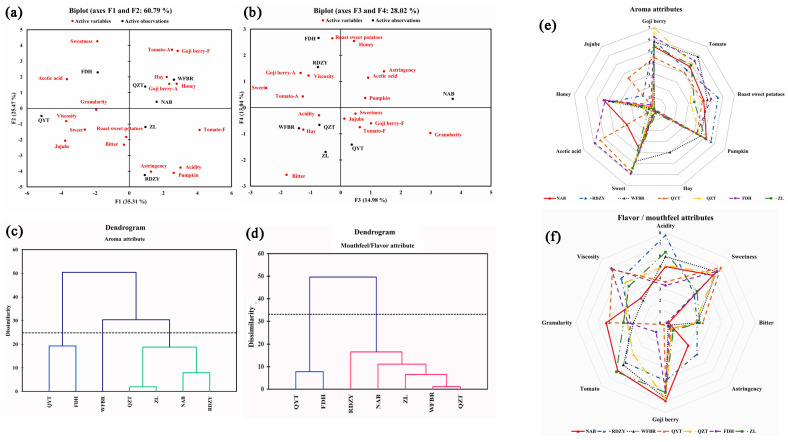
The principal component analysis, hierarchical clustering analysis of sensory attribute, and sensory profile analysis of goji berry pulp. (**a**) PC1 and PC2 of the principal component analysis for attributes of goji berry pulp (in Goji berry −A and Tomato −A, the ‘A’ stands for the abbreviation of aroma, in Goji berry −F and Tomato −F, the ‘F’ stands for the abbreviation of flavor); (**b**) PC3 and PC4 of the principal component analysis for attributes of goji berry pulp, (black font indicates samples, red font indicates sensory attributes); (**c**) clustering diagram for aroma attributes (different colors represent different aroma categories); (**d**) clustering diagram for mouthfeel/flavor attributes (different colors represent different flavor /mouthfeel categories); (**e**) goji berry pulp aroma profile chart; (**f**) goji berry pulp flavor/mouthfeel profile chart(spider web of sensory profile analysis of goji berry pulp based on cluster analysis).

Using the sensory lexicon of goji berry pulp mentioned above, a quantitative descriptive analysis (QDA) was carried out. The significant intensities of the 17 sensory attributes were subjected to PCA for further investigating the properties of the goji berry pulp. The F1 and F2 explained 60.79% of the total variance. As shown in Figure 1a, samples QYT and FDH were separated from WFBR, QZT, NAB, and ZL in the first principal component. The initial principal component concentrated on the aroma characteristics of “goji berry −A”, “tomato −A”, “pumpkin”, “honey”, “acetic acid”, “jujube”, “honey”, “acetic acid”, and “red dates” and the flavor/mouthfeel attributes of “acid”, “goji berry −F”, and “tomato −F”, and “viscosity” and ten other variables (Table S3). The sensory characteristics of QYT and FDH were characterized by an “acidity” in terms of aroma and a higher “viscosity” in terms of mouthfeel; WFBR, QZT, NAB, ZL, and RDZY had a strong “goji berry aroma” and “tomato flavor”. The second principal component reflected “tomato” and “pumpkin” in aroma, “sweetness”, “goji berry “, “acidity”, and “astringency” in flavor/mouthfeel information on the variation of the six indicators. Regarding its second principal component, “acidity” and “pumpkin” set RDZY apart from the other samples.

Of the total variance in Figure 1b, 28.06% was explained by F3 and F4. Although F3 and F4 were not always the most crucial, the complementary information they offered in this study was essential because of the intricacy of the samples. Data on the variation of the three indicators—“sweetness”, “bitterness, and “granularity”—were captured in the third principal component. The NAB sample demonstrated a considerable “granularity” and was distinguished from the other samples in this component. The variation of the three indicators—“roasted sweet potato”, “honey”, and “bitterness”—was represented by the fourth principal component. This suggested that in addition to the aroma of “acetic acid”, FDH also smelled strongly of “roasted sweet potato” and “honey” (Table S3). The PCA results also revealed that a single or a few sensory attributes do not represent the overall characteristics of a sample. Each sample is described by various sensory attributes, showing the complex sensory characteristics of the goji berry pulp samples. The PCA results further demonstrated the applicability of the goji berry pulp sensory lexicon by demonstrating that the descriptors in the pre-established sensory lexicon could fully describe the actual samples.

#### 2.3.2. Analysis of Goji Berry Pulp Flavor Types

Goji berry pulp samples were subjected to cluster analysis based on the QDA results for aroma attributes (Figure 1c), and the sensory attribute profile was drawn in Figure 1e. QZT, ZL, NAB, and RDZY were among the first group of goji berry pulp samples that showed vital sensory attributes of “goji berry”, “tomato”, “roasted sweet potatoes”, “pumpkin”, “sweet”, and “honey”, with weaker attributes of “hay” and “acetic acid” that were classified as sweet. The second group, which included QYT and FDH, was characterized by more vital “acetic acid” attributes and weaker “jujube” attributes. The third group, WFBR, was distinguished as hay by a significant “hay” attribute.

Goji berry pulp samples were also subjected to cluster analysis (Figure 1d) based on the results of the QDA for flavor/mouthfeel attributes, and their sensory attribute profile was created in Figure 1f. The flavor/mouthfeel attributes categorized the samples into two distinct groups: the first group contained five samples (RDZY, NAB, ZL, WFBR, and QZT) that exhibited evident “acidity”, with strong attributes of “goji berry” and “tomato” and moderate “sweetness”, defining them as acidic. The two samples (QYT and FDH) in the second group were classified as sweet due to their significant “sweetness” and weak “acidity” attributes.

### 2.4. The Contribution of Aroma-Active Compounds for Goji Berry Pulp

The existence of volatile compounds and their composition dictates the distinct aroma of foods and the flavor profile of the resultant products [35]. Using HS-SPME-GC-Orbitrap-MS, 83 volatile compounds were identified in goji berry pulp. These compounds included 6 acids, 10 aldehydes, 6 lactones, 12 esters, 3 ketones, 12 terpenes, 11 volatile phenols, 4 furans, 9 alcohols, 2 pyrazines, 6 benzenes, and 2 C13-nor isoprenoids (Table 4). Esters accounted for 44.22% of the volatile aroma compounds, making them the most abundant. Furans, acids, volatile phenols, alcohols, benzenes, and aldehydes accounted for 30.76%, 10.70%, 4.92%, 4.29%, 1.84%, and 1.28%, respectively. The percentage of lactones, terpenes, ketones, C13-nor isoprenoids, and pyrazines within the total volatile compounds were 0.94%, 0.71%, 0.14%, 0.03%, and 0.02%, respectively. In general, aroma compounds with OAVs ≥ 1 are considered as aroma-active in the overall aroma of the samples (Table 5). This is because they might be present in the sample at concentrations higher than their threshold values, which could cause their characteristic aroma to be expressed [36,37,38]. The following section discusses and categorizes the important volatile compounds.

#### 2.4.1. Esters

Esters play a significant role in the distinctive “fruity” and “floral” aroma attributes of fruits and juices as volatile aroma compounds [41,45]. Twelve esters were detected in the goji berry pulp and their concentrations were the highest, ranging from 0.14 to 11,508.01 μg/L. Among the ester compounds detected in goji berry pulp, e amyl acetate, isoamyl acetate, ethyl lactate, heptyl acetate, ethyl 3-hydroxybutyrate, 2-hydroxy-4-methyl ethyl valerate, and ethyl phenylacetate have not been previously reported in fresh goji berries or goji berry juice. Goji berry pulps were high in ethyl 4-methyl valerate, described as “fresh” and “blackberry”. The remaining esters were characterized as having characteristic aromatic properties such as “fruity”, “floral”, and “sweet”, and their contents were lower.

The most common ester in goji berry pulp was ethyl acetate, which ranged in concentrations from 737.00 to 2684.66 μg/L. In every sample, its OAV was greater than 1, ranging from 147.47 to 536.93. Ethyl acetate was also an important aroma substance in goji berry pulp, contributing to the “fruity” and “sweet” aromas. Another essential ester for the “fruity” and “sweet” flavor of goji juice was ethyl acetate, found in fresh goji berries and goji juice with high concentrations [46,47].

#### 2.4.2. Aldehydes

Aldehydes are prevalent volatile aroma compounds in goji berries and their products. The goji berry pulp contained ten aldehydes, all of which have a pleasant aroma. Among the aldehydes, hexanal, a common aldehyde, exhibited a “green grass” aroma and an OAV greater than 1 in five variants of goji berry juice [48]. Although hexanal was the most prevalent compound in fresh goji berries [41], goji berry pulp has less, possibly due to the high-temperature processing. The concentration range of hexanal in the sample was 3.87~45.94 μg/L. It has been demonstrated that the evaporation of hexanal during heating, concentration, and pasteurization can result in a content reduction [49]. Octanal, 1-nonanal, and *(E,E)*-2,4-heptadienal were all found in ZL, and their OAVs were greater than 1. Octanal has a “fruity” and “lemon” aroma. Aldehydes have a “fruity” and “floral” aroma, but they can also have a “fatty” aroma depending on the concentration when the chain length of the aldehyde compound increases above C6. The lipoxygenase-catalyzed oleic acid in the fruit resulted in nonanal, which has a “grassy”, “floral”, and “fatty” aroma. It has been demonstrated that because nonanal has a lower aroma threshold, it has a more significant impact on the overall aroma of the juice [50]. The aromas of *(E,E)*-2,4-heptadienal were “nutty” and “fatty”. The compound *(E,Z)*-2,6-nonadienal has the aroma of “green grass” and “cucumber” and had an OAV greater than 1 in all samples. Benzene acetaldehyde, an aldehyde with an aromatic ring, was the most prevalent aldehyde in goji berry pulp, with an OAV greater than 1 in all samples, with aromas of “floral”, “rose”, and “cherry”.

#### 2.4.3. Lactones

Lactones are also known as cyclic esters or intramolecular esters, which are aromatic compounds formed from the corresponding hydroxy acids. Six distinct lactone aroma compounds were identified in the seven samples of goji berry pulp. Of all the lactones, *δ* -hexalactone (L1), *γ*-octanoic lactone (L2), and 5-hydroxyoctanoic acid lactone (L3) were the main components, accounting for more than 90% of the total lactones. The concentration ranges were 0.06~36.72 μg/L, 3.49~8.23 μg/L, and 1.62~19.12 μg/L, respectively. Previous studies have not detected the above lactones in the goji berries and their products [39,46].

Of all the lactones, *γ*-Octanoic lactone had an OAV value greater than 1 in WFBR and *γ*-decalactone had an OAV value greater than 1 in QYT and WFBR. These two lactones contributed mainly to the pleasant aroma of “peach, cream, and coconut”. *γ*-Octanoic lactone and *γ*-decalactone were frequently detected in apricots and their products and coconut milk and cream [51,52]. The flavor and aroma of *γ*-octanoic lactone were typically described as “coconut”, “sweet”, “creamy”, and “fatty” flavor [52,53].

#### 2.4.4. Terpenoids

Many fruits and herbs have a distinct aroma that comes from terpenoids. The goji berry pulp contains 12 terpenoids. The formation of linalool, a monoterpene product in the fruit, indicates fruit ripening. The amount of linalool found in fresh wolfberry fruit was higher than in goji berry pulp, possibly because the latter was processed at a higher temperature. Heat treatment also resulted in a comparable reduction in linalool level in the study of tomato juice aroma [54]. Trans-rose oxide smelled like “floral”, “rose”, and “cherry”, and in samples WFBR and ZL, its OAV was more than 1.

#### 2.4.5. Volatile Phenols

Eleven volatile phenolic compounds were found in goji berry pulp; 4-hydroxy-3-methoxystyrene and 4-hydroxystyrene had an OAV greater than 1 in every sample, and in half of the goji berry pulp samples, eugenol (“clove” and “honey”), 3-ethylphenol (“musty”), and guaiacol (“smoky”) were able to reach the aroma threshold.

#### 2.4.6. Alcohol

Foods that are high in alcohol have been shown to produce fruit flavors and are crucial in producing flavors [36,46]. Studies on the goji berry and its products have also revealed that alcohols were the primary aroma compounds of goji berry pulp [36,39]. The most prevalent alcohol in goji berry pulp was phenylethyl alcohol, which smells like “rose” and “citrus”. Its concentration ranges from 58.83~102.18 μg/L. In the study of the aroma of fresh goji berry and its juice, 2-methyl-1-propanol, 1-pentanol, 3-methyl-1-butanol, 2-heptanol, 1-octen-3-ol, 2,3-butanediol, and 1-octanol with phenylethyl alcohol were found and their olfactory contributions were reported [47]. Only cis-6-nonen-1-ol, on the other hand, had an OAV greater than 1, except for sample FDH, which had an OAV ranging from 1.48 to 84.68 and a “fresh”, “cucumber”, and “melon” aroma. It smells “fresh”, “cucumber”, and “melon”, and reports of melon aroma are common [55].

### 2.5. Relationship between Volatile Aroma Compounds, Aroma-Active Compounds, and Aroma Attributes

**Figure 2:**
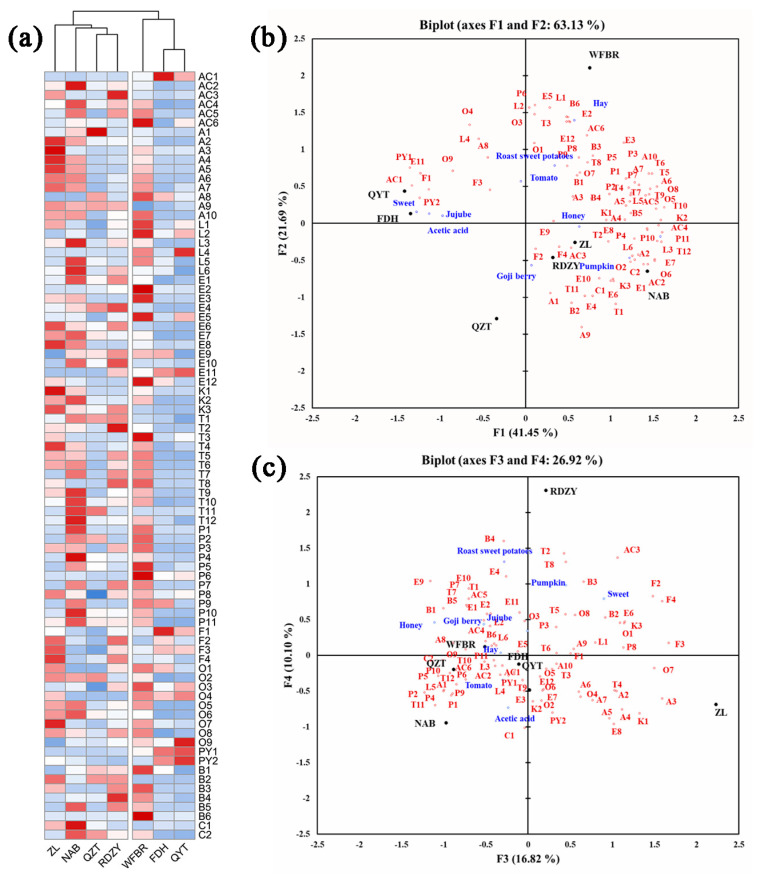
(**a**) Heat map of the volatile aroma content of the goji berry pulp. (Alphabetical symbols indicate aroma compounds, Table 3). (**b**,**c**) The correlation analysis of aroma compounds and aroma descriptors in goji berry pulp, the PCA of aroma compounds supplemented with aroma intensity of aroma descriptors. (Alphabetical symbols indicate aroma compounds, Table 3).

It is evident from the heat map of volatile aroma compounds in Figure 2a that the various goji berry pulp samples have diverse volatile aroma compound types and contents. While volatile phenols, terpenes, esters, and lactones were more abundant in NAB, RDZY, and WFBR samples, aldehydes and acids were relatively more abundant in ZL, NAB, and WFBR samples. It was discovered that the FDH and QYT samples had higher pyrazines levels.

The findings of the sensory analysis were added as additional variables to the PCA of aroma compounds to investigate potential relationships between aroma compounds and sensory qualities, as shown in Figure 2b,c.

The PCA results showed that QYT and FDH exhibit “sweet”, “jujube”, and “acetic acid” attributes. These attributes could be associated with compounds such as 2,3-dimethyl-5-ethylpyrazine (PY1), acetylpyrazine (PY2), phenylethyl alcohol (O9), butyric acid (AC1), furfural (F1), benzeneacetic acid, and ethyl ester (E11). ZL and RDZY exhibited “goji berry” and “honey” attributes. These attributes could be associated with compounds such as hexanal (A1), 2-acetylfuran (F2), furfuryl alcohol (F4), 2-ethylhexanoic acid (AC3), 2-hydroxy-4-methyl ethyl valerate (E9), octanal (A3), o-cymene (B4), 1-octen-3-one (K1), and terpinolene (T2). NAB exhibited the “pumpkin” attribute. This attribute could be associated with compounds such as caprylic acid methyl ester (E6), damascenone (C1), 2-nonanone (K3), 1-pentanol (O2), trans-2-hexenal (A2), and *δ*-dodecalactone (L6). WFBR exhibits a “hay” attribute, which could be associated with compounds such as *δ*-hexalactone (L1), 2-phenoxyethanol (B6), amyl acetate (E2), trans-rose oxide (T3), and benzoic acid (AC6). “Sweet potatoes” and “tomatoes” were likely to be associated with o-xylene (B1), 2-methyl-1-propanol (O1), *(E,Z)*-2,6-nonadienal (A8), 5-methyl furfural (F3), 3-ethylphenol (P9), 4-ethylphenol (P8), and palmitic acid ethyl ester (E12).

PLSR was used to determine the correlation between volatile aroma compounds and aroma attributes in the samples. The intensity of the aroma attributes served as the Y variable, while the concentration of the 83 volatile aroma compounds in the goji berry pulp served as the X variable. Variables with VIP values greater than 0.8 were used to develop a PLSR model [56], and the correlation coefficients are shown in Table S5.

**Figure 3:**
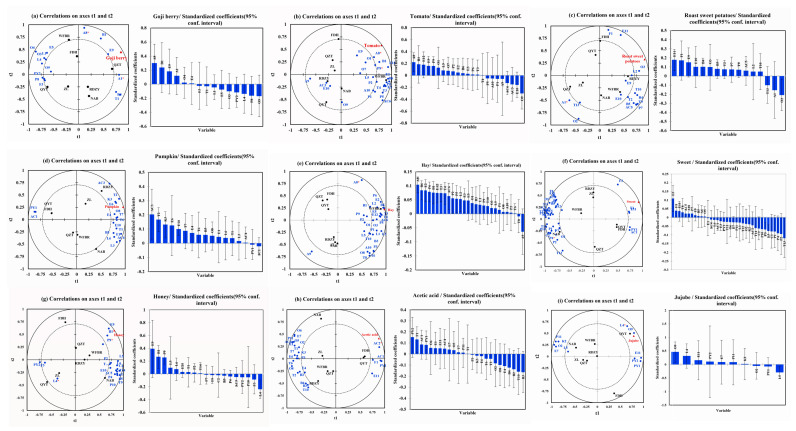
PLSR analysis of aroma attribute and volatile aroma compounds. (**a**) PLSR analysis of “goji berry aroma” and volatile aroma compounds. (“*” Indicates compound OAV > 1) (**b**) PLSR analysis of “tomato aroma” and volatile aroma compounds (“*” indicates compound OAV > 1). (**c**) PLSR analysis of “roast sweet potatoes aroma” and volatile aroma compounds (“*” Indicates compound OAV > 1). (**d**) PLSR analysis of “pumpkin aroma” and volatile aroma compounds. (**e**) PLSR analysis of “hay aroma” and volatile aroma compounds (“*” indicates compound OAV > 1). (**f**) PLSR analysis of “sweet aroma” and volatile aroma compounds (“*” indicates compound OAV > 1). (**g**) PLSR analysis of “honey aroma” and volatile aroma compounds (“*” indicates compound OAV > 1). (**h**) PLSR analysis of “acetic acid aroma” and volatile aroma compounds (“*” indicates compound OAV > 1). (**i**) PLSR analysis of “jujube aroma” and volatile aroma compounds (“*” indicates compound OAV > 1). Red indicates aroma attributes, blue indicates aroma compounds.

The chemical variables in the “goji berry” model predicted 92.06% of the variability Figure 3a. The intensity of “goji berry” in goji berry pulp was found to be positively correlated with compounds such as *(E,Z)*-2,6-nonadienal (A8), o-xylene (B1), 2-hydroxy-4-methyl ethyl valerate (E9), and hexanal (A1), whereas it was negatively correlated with concentrations of phenylethyl alcohol (O9), 4-ethylphenol (P8), *γ*-decalactone (L4), and acetylpyrazine (PY2). *(E,Z)*-2,6-Nonadienal exhibits “green grass” and “cucumber”, o-xylene exhibits “sweet” and “floral”, 2-hydroxy-4-methyl ethyl valerate exhibits “fresh” and “blackberry”, and hexanal exhibits “apple-like”, “green grass”, and “citrus”. Research has shown that hexanal is the primary compound in fresh goji berries, and a study on the aroma of fermented goji berry juice found that hexanal was positively correlated with the “goji berry” [22].

As shown in Figure 3b, the chemical variables in the “tomato” model show a predictive capacity of 90.50% of the variability. The strength of “tomato” in goji berry pulp was positively correlated with several chemicals, including *(E,Z)*-2,6-nonadienal (A8), o-xylene (B1), palmitic acid ethyl ester (E12), 2-hydroxy-4-methyl ethyl valerate (E9), 3-ethylphenol (P9), and 2-methyl-1-propanol (O1). On the other hand, there was an inverse relationship between this intensity and the amount of benzoic acid (AC6), 2-ethylhexanoic acid (AC3), methyl salicylate (E10), and phenylethyl alcohol (O9). The compounds that show a positive correlation with the “tomato” attribute include *(E,Z)*-2,6-nonadienal, which has a “green grass” and “cucumber” characteristic; o-cymene, which adds a “sweet” and “floral” characteristic; palmitic acid ethyl ester, which adds a “fruity” and “milky” characteristic; 2-hydroxy-4-methyl ethyl valerate, which is linked to a “fresh” and “blackberry” characteristic; 3-ethylphenol, which offers a “sweet” taste; and 2-methyl-1-propanol, which provides an “alcohol” taste. Benzoic acid, on the other hand, has a “fatty” correlation with the word “tomato,” and 2-ethylhexanoic acid has a “cheese” correlation.

In the “roast sweet potatoes” model, the chemical variables explained 94.35% of the variability, as shown in Figure 3c. The intensity of “roast sweet potatoes” in goji berry juice was found to be negatively correlated with hexanal (A1) and 1-pentanol (O2) and positively correlated with compounds like furfural (F1), 2-hydroxy-4-methyl ethyl valerate (E9), methyl salicylate (E10), and benzeneacetic acid. Among the substances that have a positive correlation with “roast sweet potatoes”, benzeneacetic acid ethyl ester has a “sweet”, “floral”, and “honey”, and a “sweet” furfural. Furfural, a by-product of the Maillard reaction during sweet potato baking, contributes to the “sweet” and “caramel” characteristics of baked sweet potatoes in a study of their physicochemical characteristics [50]. Conversely, 1-pentanol is “greasy” among the compounds negatively correlated with “roast sweet potatoes”. On the other hand, hexanal has an “apple-like”, “green grass”, and “citrus” aroma.

The chemical variables in the “pumpkin” model explained 98.14% of the variability seen in Figure 3d. The results of our study showed that the presence of “pumpkin” in goji berry puree juice was positively correlated with the concentrations of specific compounds, such as 2-ethylhexanoic acid (AC3), terpinolene (T2), 2-nonanone (K3), and caprylic acid methyl ester (E6). Notably, 2-ethylhexanoic acid contributed a “cheese” flavor among the chemicals positively linked with “pumpkin”. Terpinolene showed a “fresh” and “sweet” flavor as well as a “cheese” and “fruity” flavor, and caprylic acid methyl ester contributed a “fruity” flavor. Similarly, 98.34% of the variability was predicted by the chemical variables in the “hay” model (Figure 3e). Our research showed that the compound *(E,Z)*-2,6-nonadienal (A8) and the intensity of “hay” in goji berry pulp were positively correlated. This specific compound is well-known for its “green grass” and “cucumber” aromas and can be found in various tea leaves. The function of *(E,Z)*-2,6-nonadienal in increasing the aroma profile associated with green tea has been addressed in studies by Chen et al. [57] and Zheng et al. [58], stressing its contribution to the green characteristics of tea.

In the “sweet” model, the chemical variables predicted 97.30% of the variation seen in Figure 3f. The concentration of compounds such as 5-methyl furfural (F3), which has a “creamy” character, was found to be positively correlated with the intensity of “sweet” in goji berry pulp. However, the compounds geranic acid (T11), which has a “green” flavor, 4-hydroxy-3-methoxystyrene (P4), which has a “caramel” flavor, and o-cresol (P2), which has “Chinese traditional medicine” and “smoky” flavor, have a negative correlation with the intensity of “sweet” in goji berry pulp. The chemical variables in the “honey” model predicted 98.55% of the variance seen in Figure 3g. The findings demonstrated a positive correlation between the concentration of compounds and their associated flavors, such as 2-hydroxy-4-methyl ethyl valerate (E9) with a “fresh” and “blackberry” flavor, o-xylene (B1) with a “sweet” and “floral” flavor, and 3-ethylphenol (P9) with a “sweet” flavor. Conversely, there is a negative correlation between the intensity of “honey” in goji berry pulp and *γ*-decalactone (L4) with a “coconut”.

The chemical variables in the “acetic acid” model predicted 94.24% of the variance Figure 3h. The intensity of “acetic acid” in goji berry pulp was found to be positively correlated with the concentration of compounds, such as butyric acid (AC1) and hexanoic acid (AC2) but negatively correlated with the concentration of compounds, such as methyl hexanoate (E4), o-cymene (B4), styrene (B2), nerol (T10), and p-cymene (B3). Short-chain fatty acids are compounds found in a variety of foods, exhibiting an “acid” aroma. Among compounds positively correlated with an “acetic acid,” butyric acid imparts an “acid”, while hexanoic acid exhibits a “cheese” flavor. The chemical variables in the “jujube” model predicted 82.22% of the variance Figure 3i. According to the study, there is a positive correlation between the concentration of compounds such as phenylethyl alcohol (O9), *γ*-decalactone (L4), benzeneacetic acid, ethyl ester (E11), and acetylpyrazine (PY2) and the intensity of “jujube” in goji berry pulp. Conversely, there is a negative correlation between the intensity of “jujube” in goji berry pulp and the concentration of undecanolactone (L5).

**Figure 4:**
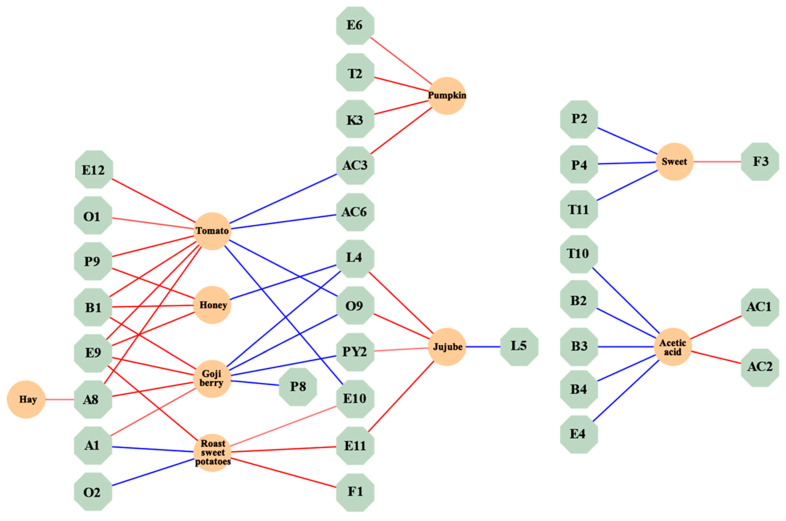
Interaction map of volatile compounds and aroma properties network. (The red connecting lines represent positive correlations, while the blue connecting lines represent negative correlations. The thicker the connecting line, the stronger the correlation).

The standardized regression coefficients of volatile compounds and aroma attributes are shown in a network diagram to explore the relationship between the two. In Figure 4, 3-ethylphenol (P9) with a “sweet” attribute was positively correlated with “tomato” and “honey” in aroma characteristics. o-Xylene (B1) with “sweet” and “floral” attributes was positively correlated with “tomato”, “honey”, and “goji berry” in aroma characteristics. 2-hydroxy-4-methyl ethyl valerate (E9) with “fresh” and “blackberry” attributes was positively correlated with “tomato”, “honey”, “goji berry”, and “roasted sweet potatoes” in aroma characteristics. *(E,Z)*-2,6-nonadienal (A8) with “green grass” and “cucumber” attributes was positively correlated with “cucumber”, “goji berry”, and “hay” in aroma characteristics. Benzeneacetic acid and ethyl ester (E11) with “sweet” and “floral” attributes were positively correlated with “roasted sweet potatoes” and “jujube” in aroma characteristics. Hexanal (A1) with “green grass” and “apple-like” attributes was positively correlated with “goji berry” in aroma characteristics. Acetylpyrazine (PY2) with a “roast” attribute was positively correlated with “jujube” in aroma characteristics. 2-Ethylhexanoic acid (AC3) with a “creamy” attribute was positively correlated with “pumpkin” in aroma characteristics. *γ*-Decalactone (L4) and phenylethyl alcohol (O9) exhibited positive correlations with the aroma characteristics of “jujube”. Additionally, methyl salicylate (E10) showed a positive correlation with the aroma characteristics of “roasted sweet potatoes”.

The findings of this section suggest that some volatile compounds will simultaneously correlate with multiple aroma attributes.

## 3. Materials and Methods

### 3.1. Sample Collection and Chemicals

This study investigated a set of seven goji berry pulp products that are sold in the Chinese market. Table S1 shows the details of the experimental samples. Ethanol and dichloromethane of HPLC grade were purchased from Honeywell (Marris Township, NJ, USA). All analytical reagents, which included tartaric acid, sodium chloride (NaCl), sodium hydroxide (NaOH), and glucose, were purchased from Beijing Chemical Works (Beijing, China). An n-alkanes solution (C6–C24) was purchased from Supelco (Bellefonte, PA, USA). Table S2 lists the authentic flavor standards purchased commercially and used for qualitative and quantitative analysis of aroma-active compounds.

### 3.2. Measurement of Basic Physical and Chemical Indicators

A pH meter model HI2221 (HANNA Instruments, Padua, Italy) was used to measure the pH values of seven goji berry pulp samples, and a digital display saccharimeter model 2818 (Spectrum, Stamford, CT, USA) was used to measure the soluble solids at room temperature. The high-performance liquid chromatography (HPLC) method previously published in our study was used to examine the organic acids, such as lactic acid, tartaric acid, oxalic acid, citric acid, and malic acid, and reducing sugars, such as glucose and fructose [52]. A Venusil XSB C18 column (4.6 mm × 250 mm, 5 μm; Bonna-Agela Technologies Co. Ltd., Tianjin, China) and a PDA detector set at 210 nm were included in the Shimadzu LC-20AT system (Shimadzu, Kyoto, Japan), which measured the concentration of organic acids. A Venusil Innova Durashell NH_2_ column (4.6 mm × 250 mm, 5 μm; Bonna-Agela Technologies Co. Ltd., Tianjin, China), a Shimadzu LC-20AT HPLC, and an RID-20A detector (Shimadzu, Japan) were used to examine reducing sugars. Each analysis was performed in triplicate for each sample.

### 3.3. Sensory Analysis

#### 3.3.1. Open-Ended Questions

Seventy-six participants (18~26 years old) from Beijing Forestry University were asked to participate in the experiment with open-ended questions. The participants were asked to use at least three words to describe the sample.

#### 3.3.2. Established of Goji Berry Pulp Descriptive Sensory Lexicon

##### Selection and Training of Sensory Panel

In accordance with the requirements of GB/T16291.1-2012(Sensory analysis--General guidance for the selection, training and monitoring of assessors--Part 1:Selected assessors), 15 evaluators (5 males, 10 females, aged 18–24 years) were selected from Beijing Forestry University. For 2 months (twice/week, 2 h/session), the evaluators received training in ISO 8586(Selection And Training Of Sensory Assessors), and the tests were administered in a controlled sensory laboratory environment. After sniffing or tasting the samples, the panelists did not experience any discomfort. The consistency of the evaluators was assessed by plotting Tucker-1 correlation loadings plots using the 9-point intensity scale for sensory evaluation; their discrimination and repeatability were evaluated using Panel Check software (version 1.4.0) to calculate the F and mean square error values.

Furthermore, 12 professional sensory evaluators (4 males, 7 females, aged 18–24 years) were selected based on their overall performance. In the later stages of the training, each evaluator could independently conduct a sensory evaluation of each attribute.

##### Selection and Development of Sensory Attributes and Reference

The first step was collecting and analyzing the descriptors subjected to the submitted open-ended questions. These descriptors were then revised and integrated following the guidelines provided in ISO 11035 (Sensory analysis—Identification and selection of descriptors for establishing a sensory profile by a multidimensional approach). Considerations included eliminating quantitative terms and personal preference expressions and merging descriptors with similar meanings. The descriptors assigned to the sensory wheel were analyzed to determine their geometric mean M and variance. Based on these results, descriptors with low M values (i.e., descriptors with low M values, meaning they contributed less to the flavor of the samples) were eliminated. The geometric mean M was calculated based on the following formula:

$$M=\sqrt{F\times I}$$

*F* is the percentage of the total number of times that the descriptor could have been stated that the descriptor was stated. *I* represents the strength of a descriptor that the evaluation team provided and the percentage of the maximum possible strength that could be obtained for that descriptor.

After the evaluation team was given the list of descriptors eliminated by the M-value method, a detailed discussion and literature review were undertaken to identify reference samples that would be appropriate for each descriptor. After the tasting, the evaluation team held a group discussion and gave the evaluator 3–5 samples to determine the reference sample of this descriptor. The panel was also provided standard aqueous solutions of 1-butanol in varying concentrations, along with the identified reference sample. The evaluators sniffed the reference sample and then selected an aqueous solution of 1-butanol with an odor intensity comparable to the reference sample. They then recorded the concentrations of butanol, which accurately mirrored the odor intensity.

#### 3.3.3. Sensory Evaluation

Panelists were randomly given seven goji berry pulps, each with a 3-digit arbitrary code. The samples were given to the evaluators in two batches, with intervals in between and a water rinse to rinse the mouth after each sample evaluation. To evaluate the sensory qualities of the samples, evaluators used a 9 cm linear scale with the left and right anchors labeled “0 (not intense)” and “9 (very intense)”, respectively. Two replicates of the sensory evaluation experiment were carried out; the second replication was completed the following day.

### 3.4. HS-SPME-GC-Orbitrap-MS Analysis of Goji Berry Pulp

#### 3.4.1. HS-SPME

In a 15 mL headspace vial with polytetrafluoroethylene (PTFE) septum, goji berry pulp sample (5 mL), NaCl (1.00 g), and 10 μL 4-methyl-2-pentanol (1.077 g/L) were mixed. Once the sample in the vial had been equilibrated on the heating and agitation platform for 30 min, using 60 °C as the equilibration/sorption temperature, the fiber was placed into the headspace of the vial to sorb the volatiles for an additional 30 min. Upon extraction, the extraction head was removed and immediately inserted into the GC inlet, where it was revolved at 250 °C for 10 min.

#### 3.4.2. GC-Orbitrap-MS Analysis System

Analytes were separated using a VF-WAXms column (60 m × 0.25 mm × 0.25 μm, J&W Scientific, Folsom, CA, USA) with a 1.2 mL/min flow rate under carrier gas (helium, 99.999% purity). The chromatographic temperature rise was as follows: kept at 40 °C for 5 min, then the temperature was increased to 180 °C at a speed of 3 °C/min, and then to 250 °C at a speed of 3 °C /min. In the Orbitrap MS, the positive ion electron ionization was set at 70 eV, and the ion source temperature was 280 °C. The temperature of the MS interface is 230 °C. MS acquisition was carried out in profile mode using a *m*/*z* range of 30–330 *m*/*z*.

#### 3.4.3. Qualitative and Quantitative Analysis

Retention indices were calculated with an alkane series spanning from C6 to C24 (Supelco, Bellefonte, PA, USA). By comparing retention indices of the volatile aroma compounds in the pulp of goji berries and their MS with a high-resolution MS database established in our laboratory (http://foodflavorlab.cn/, accessed on 20 June 2023), we could qualitatively analyze the compounds. Depending on the concentration of the volatiles in the goji berry pulp, different volatile standards were dissolved in a goji berry pulp solution and then sequentially diluted into 15 gradients for quantitative analysis. The analytical determination was carried out twice for each gradient using Trace Finder 4.1 (Thermo Fisher Scientific, Les Ulis, France), adhering to the guidelines outlined in Section 3.4.2. The findings were deconvoluted and processed using the Processing setup, Quan Browser, and Qual Browser features of Xcalibur version 4.1 software [59]. Target compounds underwent quantitative analysis in which the concentration and peak area ratio of the compound were determined to create a standard curve. The standard curve with the same number of carbon atoms or a similar functional structure was used for quantification when no standard was available for volatile compounds.

#### 3.4.4. Odor Activity Value

According to current studies, the contribution of aroma compounds is assessed using OAVs [24]. The OAV was calculated by dividing the odorant concentration by the corresponding orthonasal odor threshold [60]. The odor thresholds for various compounds were established based on the relevant literature.

### 3.5. Statistical Analysis

The XLSTAT 19 (Addinsoft, New York, NY, USA) was used to build agglomerative hierarchical clustering, PCA, and PLSR. Tukey’s post hoc test was used to compare significant differences between means in a one-way analysis of variance using R 3.6.3. Statistical significance was determined at α = 0.05. Microsoft Office 2019 was used to create the radar chart and bar chart.

## 4. Conclusions

In this study, the fundamental physicochemical characteristics of goji berry pulp were examined, and a sensory lexicon relevant to this product was created together with selected attribute reference samples. Nine terms were associated with the aroma, including “goji berry”, “tomato”, “roast sweet potato”, “pumpkin”, “hay”, “sweet”, “acetic acid”, “honey”, and “jujube”, and eight terms, “acidity”, “sweet”, “bitter”, “astringent”, “goji berry”, “tomato”, “granularity”, and “viscosity”, were associated with the flavor/mouthfeel. According to the established sensory lexicon, the organoleptic characteristics were revealed by QDA, and they were categorized into sweet, acetic, and hay types according to their aroma attributes. A total of 83 volatile aroma compounds were identified in goji berry pulp using HS-SPME-GC-Orbitrap-MS technology. Using OAV in combination, we discovered that 17 aroma-active compounds were the essential components of goji berry pulp. Among them, hexanoic acid, *(E,Z)*-2,6-nonadienal, benzeneacetaldehyde, ethyl acetate, 4-hydroxy-3-methoxystyrene, and 4-hydroxystyrene had OAVs greater than 1 in all the goji berry pulp samples. Based on the PCA of volatile aroma compounds and aroma attributes, as well as the PLSR model, it was found that compounds such as 3-ethylphenol, methyl octanoate, 2-hydroxy-4-methyl ethyl valerate, ethyl phenylacetate, hexanal, *(E,Z)*-2,6-nonadienal, acetylpyrazine, butyric acid, 2-ethylhexanoic acid, 2-methyl-1-propanol, 1-pentanol, phenylethyl alcohol, and 2-nonanone might play a significant role in contributing to the aroma of the goji berry pulp. Future research could further explore the evolution of important sensory attributes and related flavor compounds in goji berry pulp during processing and storage, providing a basis for regulating the production and quality control of goji berry pulp.

## Figures and Tables

**Table 1:** Contents of reducing sugar, organic acid, pH, and soluble solids in goji berry pulp.

Compound	Sample							
	FDH	QYT	QZT	NAB	RDZY	ZL	WFBR	Significant
***Organic acids* (g/L)**								
Oxalic acid	0.17 ± 0.00 ^f^	0.10 ± 0.04 ^g^	0.38 ± 0.00 ^d^	0.73 ± 0.01 ^a^	0.52 ± 0.00 ^c^	0.58 ± 0.01 ^b^	0.24 ± 0.00 ^e^	**
Tartaric acid	1.60 ± 0.03 ^e^	1.44 ± 0.00 ^f^	2.07 ± 0.00 ^b^	2.16 ± 0.01 ^a^	1.93 ± 0.00 ^c^	1.74 ± 0.01 ^d^	1.40 ± 0.07 ^f^	**
Malic acid	3.44 ± 0.01 ^c^	3.01 ± 0.00 ^d^	3.37 ± 0.00 ^c^	2.39 ± 0.01 ^e^	5.20 ± 0.14 ^a^	4.73 ± 0.01 ^b^	1.80 ± 0.21 ^f^	**
Lactic acid	1.35 ± 0.00 ^b^	1.06 ± 0.00 ^c^	1.28 ± 0.00 ^b^	1.14 ± 0.01 ^c^	1.34 ± 0.06 ^b^	1.36 ± 0.14 ^b^	3.34 ± 0.01 ^a^	**
Citric acid	3.14 ± 0.01 ^f^	3.54 ± 0.00 ^e^	6.82 ± 0.00 ^c^	8.37 ± 0.03 ^a^	7.63 ± 0.14 ^b^	5.27 ± 0.14 ^d^	5.39 ± 0.00 ^d^	**
***Reducing sugars* (g/L)**								
Fructose	59.71 ± 3.44 ^c^	50.04 ± 4.17 ^c^	82.04 ± 4.55 ^ab^	92.59 ± 3.75 ^ab^	89.39 ± 1.79 ^ab^	96.60 ± 13.62 ^a^	78.62 ± 9.91 ^b^	**
Glucose	33.33 ± 5.03 ^bc^	28.00 ± 4.21 ^c^	51.34 ± 3.41 ^a^	52.18 ± 0.50 ^a^	49.62 ± 6.57 ^a^	53.70 ± 1.47 ^a^	38.33 ± 1.10 ^b^	**
***pH***								
pH	4.24 ± 0.01 ^b^	4.38 ± 0.02 ^a^	4.41 ± 0.01 ^a^	4.37 ± 0.04 ^a^	4.00 ± 0.02 ^c^	4.25 ± 0.03 ^b^	4.27 ± 0.00 ^b^	**
***Soluble solids***								
Soluble solids	13.13 ± 0.06 ^f^	12.83 ± 0.06 ^g^	15.37 ± 0.06 ^e^	18.60 ± 0.10 ^b^	15.73 ± 0.06 ^d^	20.70 ± 0.01 ^a^	17.23 ± 0.35 ^c^	**

Data are mean ± standard deviation of triplicate tests; different letters indicate significant differences; significant level: ** *p* < 0.01.

**Table 2:** Geometric mean M of goji berry pulp.

Aroma	M ^1^	Flavor/Mouthfeel	M ^1^	Flavor/Mouthfeel	M ^1^
**Goji berry**	**0.609**	**Sweetness**	**0.588**	**Granularity**	**0.430**
**Tomato**	**0.571**	**Acidity**	**0.555**	Smooth	0.193
**Pumpkin**	**0.397**	**Bitter**	**0.339**	Turbidity	0.185
Hawkthorn	0.195	**Astringency**	**0.283**	Precipitation	0.016
Carrot	0.116	**Goji berry**	**0.471**	Homogeneity	0.169
Floral	0.179	Hawkthorn	0.126		
**Roast sweet potatoes**	**0.514**	**Tomato**	**0.394**		
Fruity	0.143	Sweet potatoes	0.197		
**Sweet**	**0.634**	Carrot	0.163		
**Acetic acid**	**0.207**	Jujube	0.177		
Chinese traditional medicine	0.098	Fermented	0.023		
Fermented	0.128	Alcohol	0.006		
Spicy	0.036	Herbal	0.103		
**Honey**	0.406	Burnt taste	0.185		
Preserved fruit	0.063	Fruity	0.127		
Plum candy	0.083	Nutty	0.056		
Soy sauce	0.119	Fruit vinegar	0.167		
Tea	0.063	Pickle	0.013		
**Hay**	0.254	Creaminess	0.115		
Argy Wormwood	0.034	Herbaceous	0.141		
Nutty	0.027	Citrus	0.002		
Plant	0.126	Tangerine Peel	0.212		
Loquat leaves	0.143	Cough syrup	0.068		
Grain	0.083	Flat	0.127		
**Jujube**	0.246	**Viscosity**	0.680		

^1^ M is the geometric mean of the product of F and I. (Sensory attributes with M values greater than 0.200 are bolded in the table).

**Table 3:** Goji berry pulp sensory attributes, reference, and definitions.

Attributes	Reference Sample ^1^	Definitions ^2^	Intensity ^3^
Aroma			
Goji berry	Dried goji berries rehydrated and juiced	The aroma of goji berries.	7.0
Tomato	Fresh cherry tomato	The aroma of fresh baby tomatoes.	5.0
Roast sweet potatoes	Steam dried sweet potatoes	The sweet and tangy flavor of sweet potatoes.	6.0
Pumpkin	Steamed pumpkin	The sweet aroma of pumpkin after it has been steamed.	7.0
Hay	Dry wheatgrass	Aromas similar to those of sun-dried grasses, dry and without water.	5.0
Sweet	Dissolved brown sugar in hot water	The aroma produced by sweet substances such as marshmallows, caramel, vanilla, or sweet fruit flavored sweets (not berries).	4 g/100 g = 5.0 10 g/100 g = 8.0
Acetic acid	Apple cider vinegar ^a^	Strong aromas associated with sour products.	5 g/100 g = 5.0 10 g/100 g = 9.0
Honey	Honey dissolved in warm water	Sweet aroma reminiscent of sugar or honey.	5 g/100 g = 4.0 10 g/100 g = 6.0
Jujube	Dried jujube ^b^	The aroma of sun-dried red dates.	7.0
Flavor/mouthfeel			
Acidity	Citric acid solution	Complex taste sensations generally due to the presence of organic acids.	0.015% = 2.0 0.05% = 5.0 0.1% = 9.0
Sweetness	Sucrose solution	A basic taste produced by dilute aqueous solutions of natural or artificial substances such as sucrose or aspartame.	2.0% = 2.0 5.0% = 5.0 8.0% = 9.0
Bitter	Quinine solution	A basic taste produced by dilute aqueous solutions of substances such as quinine, caffeine, etc.	0.01% = 2.0 0.03% = 5.0
Astringency	Alum solution	The chemical feeling factor on the tongue described as puckering/drying and associated with tannins or alum.	0.01% = 2.0 0.07% = 5.0
Goji berry	Dried goji berries rehydrated and then juiced	After entering the mouth, the back of the nose smells of goji berries.	9.0
Tomato	Tomato sauce ^c^	After entering the mouth, the back of the nose smells of tomatoes.	9.0
Granularity	Peach juice ^d^; orange juice ^e^	Geometric texture properties related to the size, shape, and number of particles perceived in the product.	Peach juice = 2.0 Orange juice = 9.0
Viscosity	Mango juice ^f^; yogurt ^g^	High liquid viscosity is associated with mechanical texture properties that prevent mobility; high liquid viscosity is rich in contents; low viscosity is associated with low mechanical texture properties that prevent mobility.	Mango juice = 5.0 Yogurt = 9.0

^1^ Selected physical reference samples: ^a^ HaiTian^®^ Apple Cider Vinegar, ^b^ ZaoXiangCun ^®^ Dried dates, ^c^ HEINZ ^®^Ketchup, ^d^ HuiYuan ^®^Peach juice, ^e^ The Coca-Cola Company ^®^ Minute Maid Orange, ^f^ Nongfu Spring ^®^ Mango juice, ^g^ JUNLEBAO^®^ Flavored fermented milk; ^2^ References [16,33,34], GB/T 10221-2021 Sensory Analysis-Vocabulary; ^3^ Intensity, sensory attribute intensity on a 9 cm linear scale, aqueous solutions of 1-butanol at different concentrations as odor intensity referencing scales.

**Table 4:** The qualitative information of analyzed volatile compounds in the goji berry pulp.

Number	Compounds	RI ^1^	Identification ^2^	RT ^3^	CAS	Standard Curve	R^2^	Linear Range
								(µg/L)
AC1	Butyric Acid	1647	NIST, St, RI	39.603924	107-92-6	y = 111.75x − 0.005	0.9959	0.75–193.50
AC2	Hexanoic acid	1862	NIST, St, RI	47.584073	142-62-1	y = 6.0488x + 0.0006	0.9983	2.51–1289.00
AC3	2-Ethylhexanoic acid	1963	NIST, St, RI	51.033006	149-57-5	y = 0.2256x + 0.0009	0.9944	0.24–31.53
AC4	Octanoic acid	2075	NIST, St, RI	53.64216	124-07-2	y = 0.8824x + 0.0012	0.9949	1.38–88.87
AC5	Decanoic acid	2283	NIST, St, RI	56.96535	334-48-5	y = 0.4348x − 0.0009	0.9939	0.98–7.87
AC6	Benzoic acid	2517	NIST, RI	61.209156	65-85-0	y = 3.0379x + 0.1641	0.9819	1.13–1163.00
A1	Hexanal	1086	NIST, St, RI	14.585943	66-25-1	y = 2.5801x + 0.0014	0.9922	0.34–87.50
A2	Trans-2-Hexenal	1214	NIST, St, RI	20.979242	6728-26-3	y = 2.2493x − 0.0003	0.9971	0.17–11.12
A3	Octanal	1291	NIST, St, RI	24.298557	124-13-0	y = 1.427x − 0.0006	0.9998	0.05–14.62
A4	*(E)*-Hept-2-enal	1326	NIST, St, RI	25.934949	18829-55-5	y = 0.3582x − 0.0002	0.9982	0.15–5.10
A5	1-Nonanal	1396	NIST, St, RI	29.086784	124-19-6	y = 0.5192x − 0.0023	0.9932	0.38–12.18
A6	*(E)*-2-Octenal	1432	NIST, St, RI	30.648623	2548-87-0	y = 0.2236x + 3 × 10^−5^	0.9987	0.11–1.85
A7	*(E,E)*-2,4-Heptadienal	1497	NIST, St, RI	33.478474	881395	y = 1.5767x + 0.0005	0.9976	1.24–19.96
A8	*(E,Z)*-2,6-Nonadienal	1577	NIST, St, RI	36.815138	557-48-2	y = 0.1447x + 3 × 10^−5^	0.9973	0.05–0.81
A9	Benzeneacetaldehyde	1651	NIST, RI	39.711703	122-78-1	y = 0.9962x − 0.0026	0.9986	0.13–143.00
A10	*(E,E)*-2,4-Decadienal	1773	NIST, RI	45.868588	25152-84-5	y = 0.1447x + 3 × 10^−5^	0.9973	0.05–0.81
L1	*δ*-Hexalactone	1721	NIST, St, RI	42.042428	823-22-3	y = 62.841x − 0.0037	0.9995	0.38–48.75
L2	*γ*-Octanoic lactone	1927	NIST, St, RI	49.780074	104-50-7	y = 0.7765x + 0.0021	0.9969	2.02–64.68
L3	5-Hydroxyoctanoic acid lactone	1979	NIST, St, RI	51.634773	698-76-0	y = 9.7651x + 0.0012	0.9962	0.42–27.18
L4	*γ*-Decalactone	2158	NIST, St, RI	54.980418	706-14-9	y = 0.1745x − 0.0006	0.9999	0.45–14.45
L5	Undecanolactone	2345	NIST, St, RI	57.715314	710-04-3	y = 3.8886x + 1 × 10^−5^	0.9986	0.32–5.27
L6	*δ*-Dodecalactone	2451	NIST, St, RI	59.821499	713-95-1	y = 2.8659x + 0.0002	0.9994	0.49–7.89
E1	Ethyl acetate	892	NIST, St, RI	6.4504052	141-78-6	y = 79.05x + 0.1993	0.9929	2.50–5128.5
E2	Amyl acetate	1124	NIST, RI	16.455517	628-63-7	y = 104.76x − 0.0011	0.9949	1.00–32.00
E3	Isoamyl acetate	1125	NIST, St, RI	16.514281	123-92-2	y = 0.3106x − 5 × 10^−6^	0.9967	0.27–4.44
E4	Methyl hexanoate	1189	NIST, St, RI	19.490384	106-70-7	y = 104.76x − 0.0011	0.9949	1.00–32.00
E5	Ethyl lactate	1346	NIST, St, RI	26.845246	97-64-3	y = 25.118x − 0.0004	0.9999	1.24–19.84
E6	Methyl octanoate	1391	NIST, St, RI	28.803338	111-11-5	y = 0.0525x − 1 × 10^−5^	0.9998	0.04–1.56
E7	Heptyl acetate	1375	NIST, St, RI	31.435109	112-06-1	y = 0.299x − 7 × 10^−5^	0.9988	0.04–2.56
E8	Ethyl 3-hydroxybutyrate	1522	NIST, St, RI	38.265666	5405-41-4	y = 111.75x − 0.005	0.9959	0.75–193.50
E9	2-hydroxy4-methyl ethyl valerate	1567	NIST, St, RI	36.334623	10348-47-7	y = 18.929x + 0.0558	0.9977	0.24–1018.00
E10	Methyl salicylate	1783	NIST, St, RI	44.741398	119-36-8	y = 0.0491x + 0.0002	0.9922	0.11–3.71
E11	Ethyl phenylacetate	1789	NIST, St, RI	44.992882	101-97-3	y = 0.0359x + 1 × 10^−5^	0.999	0.02–0.37
E12	Palmitic acid ethyl ester	2246	NIST, St, RI	56.45789	628-97-7	y = 0.9081x − 0.0003	0.9961	0.24–3.89
K1	1-Octen-3-one	1303	NIST, St, RI	24.862106	4312-99-6	y = 0.4629x + 5 × 10^−6^	0.9943	0.37–6.05
K2	6-Methyl-5-heptene-2-one	1340	NIST, RI	26.515875	110-93-0	y = 0.2877x − 0.001	0.9984	0.04–47.25
K3	2-Nonanone	1390	NIST, St, RI	28.798847	821-55-6	y = 0.333x − 2 × 10^−5^	0.9998	0.09–5.80
			NIST, St, RI					
T1	(+)-Dipentene	1200	NIST, St, RI	20.009219	5989-27-5	y = 0.6026x − 6 × 10^−5^	0.9987	0.04–1.45
T2	Terpinolene	1286	NIST, St, RI	24.018626	586-62-9	y = 1.079x − 1 × 10^−5^	0.999	0.05–0.91
T3	Trans-rose oxide	1354	NIST, St, RI	27.174766	876-18-6	y = 0.0816x − 0.0006	0.9968	0.10–26.12
T4	Citronellal	1468	NIST, RI	34.098204	106-23-0	y = 0.3195x + 9 × 10^−6^	0.9945	0.04–0.76
T5	Linalool	1547	NIST, St, RI	35.530769	78-70-6	y = 0.0973x + 5 × 10^−6^	0.9994	0.05–1.88
T6	Citronellyl acetate	1666	NIST, St, RI	40.353888	150-84-5	y = 0.0426x + 0.0002	0.9967	0.03–4.96
T7	Citronellol	1690	NIST, St, RI	41.225103	106-22-9	y = 0.3195x + 9 × 10^−6^	0.9945	0.04–0.76
T8	*α*-Terpineol	1699	NIST, St, RI	41.588857	98-55-5	y = 0.1135x − 0.0005	0.9968	0.04–1.58
T9	Citral	1735	NIST, St, RI	42.967532	5392-40-5	y = 0.1371x + 7 × 10^−6^	0.9992	0.02–0.41
T10	Nerol	1802	NIST, St, RI	47.346061	106-25-2	y = 0.1062x + 0.0027	0.9926	0.15–77.5
T11	Geranic acid	2308	NIST, St, RI	57.445866	459-80-3	y = 0.5129x + 0.0015	0.9943	0.01–20.25
T12	Farnesol	2364	NIST, St, RI	58.492222	4602-84-0	y = 0.7456x + 0.0001	0.9914	0.10–1.60
P1	2-Methoxy-4-methylphenol	1966	NIST, St, RI	51.104859	93-51-6	y = 0.6115x + 0.0023	0.9925	4.81–77.10
P2	o-Cresol	2016	NIST, St, RI	52.550895	95-48-7	y = 0.6801x + 0.0006	0.9994	1.21–19.45
P3	4-Ethyl-2-methoxyphenol	2043	NIST, St, RI	53.026919	2785-89-9	y = 0.1503x + 0.0038	0.9914	3.14–100.78
P4	4-Hydroxy-3-methoxystyrene	2087	NIST, RI	55.815706	7786-61-0	y = 0.1503x + 0.0038	0.9914	3.14–100.78
P5	p-Cresol	2093	NIST, St, RI	53.98346	106-44-5	y = 1.0405x + 0.0051	0.9841	2.78–44.53
P6	m-Cresol	2101	NIST, St, RI	54.118184	108-39-4	y = 1.0405x + 0.0051	0.9841	2.78–44.53
P7	Eugenol	2177	NIST, RI	55.317228	97-53-0	y = 0.8079x + 0.0697	0.996	4.96–1270.00
P8	4-Ethylphenol	2186	NIST, St, RI	55.474405	123-07-9	y = 0.3294x + 0.0015	0.9756	1.31–42.03
P9	3-Ethylphenol	2194	NIST, St, RI	55.595657	620-17-7	y = 0.3294x + 0.0015	0.9756	1.31–42.03
P10	4-Hydroxystyrene	2276	NIST, St, RI	59.138898	2628-17-3	y = 0.8079x + 0.0697	0.996	4.96–1270.00
P11	Guaiacol	1868	NIST, St, RI	47.795141	32994	y = 0.6115x + 0.0023	0.9925	4.81–77.10
F1	Furfural	1470	NIST, St, RI	32.33332	35796	y = 3.5373x − 0.0674	0.9982	10.25–2625.00
F2	2-Acetylfuran	1510	NIST, St, RI	34.035333	1192-62-7	y = 1.3042x − 0.0052	0.9927	1.20–153.75
F3	5-Methyl furfural	1579	NIST, St, RI	36.8825	620-02-0	y = 0.7153x − 0.0017	0.9997	0.21–108.75
F4	Furfuryl alcohol	1666	NIST, St, RI	40.326942	98-00-0	y = 75.31x + 0.0045	0.9978	1.32–676.25
O1	2-Methyl-1-propanol	1092	NIST, St, RI	15.024394	78-83-1	y = 1.7695x − 0.0004	0.9969	0.50–16.10
O2	1-Pentanol	1207	NIST, St, RI	22.402133	71-41-0	y = 14.109x + 0.0023	0.9941	0.15–79.25
O3	3-Methyl-1-butanol	1250	NIST, St, RI	20.387796	123-51-3	y = 25.281x − 6 × 10^−5^	0.9995	1.92–61.57
O4	2-Heptanol	1318	NIST, St, RI	25.583537	543-49-7	y = 0.241x − 1 × 10^−6^	0.999	0.10–1.69
O5	cis-6-Nonen-1-ol	1386	NIST, St, RI	28.610233	35854-86-5	y = 2.4103x − 0.0005	0.9988	0.05–26.12
O6	1-Octen-3-ol	1341	NIST, RI	31.439599	3391-86-4	y = 0.4629x + 5 × 10^−6^	0.9943	0.37–6.05
O7	2,3-Butanediol	1522	NIST, St, RI	34.354179	513-85-9	y = 3.4254x + 0.0021	0.9971	2.55–81.90
O8	1-Octanol	1530	NIST, St, RI	36.029247	111-87-5	y = 0.2801x − 9 × 10^−5^	0.998	0.11–1.76
O9	Phenylethyl Alcohol	1922	NIST, RI	49.726184	60-12-8	y = 1.75x + 0.0355	0.9925	79.20–1267.25
PY1	2,3-Dimethyl-5-ethylpyrazine	1461	NIST, St, RI	31.897713	15707-34-3	y = 0.1053x + 1 × 10^−5^	0.9992	0.05–0.87
PY2	Acetylpyrazine	1631	NIST, St, RI	38.966231	22047-25-2	y = 5.3403x − 6 × 10^−5^	0.9996	0.02–3.14
B1	o-Xylene	1187	NIST, St, RI	17.321873	95-47-6	y = 0.8021x + 0.0639	0.9891	0.03–608.00
B2	Styrene	1260	NIST, St, RI	22.87529	100-42-5	y = 0.1717x − 6 × 10^−5^	0.9973	0.05–0.85
B3	p-Cymene	1273	NIST, RI	23.431005	99-87-6	y = 0.0468x − 2 × 10^−5^	0.999	0.03–0.56
B4	o-Cymene	1327	NIST, RI	27.580791	527-84-4	y = 0.0468x − 2 × 10^−5^	0.999	0.03–0.56
B5	Naphthalene	1748	NIST, St, RI	43.439067	91-20-3	y = 0.0198x − 1 × 10^−5^	0.9981	0.05–0.80
B6	2-Phenoxyethanol	2021	NIST, RI	52.654184	122-99-6	y = 1.75x + 0.0355	0.9925	79.20–1267.25
C1	Damascenone	1824	NIST, RI	46.245815	23696-85-7	y = 0.0216x + 5 × 10^−5^	0.9932	0.03–1.21
C2	Irisone	1945	NIST, St, RI	50.37735	14901-07-6	y = 0.0068x + 0.0012	0.9955	0.03–19.5

^1^ Retention index of compounds on VF-WAX; ^2^ “NIST” indicates the identification of compounds based on mass spectra, “RI” indicates the identification of a compound on the basis of its retention index, “St” indicates the identification of the compound against a standard; ^3^ the RT value is the time to peak of the volatile aroma substance.

**Table 5:** OAVs of volatile compounds in the goji berry pulp samples (OAV > 1).

Classification	Compounds	Aroma Threshold ^1^	Odor Quality ^2^	FDH	NAB	QYT	QZT	RDZY	WFBR	ZL
**Acids**										
**AC2**	Hexanoic acid	35.6	Cheese	**2.48 ± 0.16 ^c3^**	**19.05 ± 4.38 ^a^**	**3.64 ± 0.11 ^c^**	**7.28 ±1.21 ^bc^**	**9.52 ± 2.57 ^b^**	**7.15 ± 0.87 ^bc^**	**9.25 ± 2.18 ^b^**
**Aldehydes**										
**A1**	Hexanal	4.5	Apple-like, green grass, citrus	0.85 ± 0.07 ^c^	**4.46 ± 0.05 ^b^**	0.81 ± 0.25 ^c^	**10.20 ± 1.69 ^a^**	**1.38 ± 0.20 ^c^**	**2.07 ± 0.02 ^c^**	**1.97 ± 0.18 ^c^**
**A3**	Octanal	0.7	Fruity, green grass, lemon	0.93 ± 0.91 ^b^	**1.19 ± 0.67 ^b^**	**1.14 ± 0.41 ^b^**	tr ^4^	0.34 ± 0.03 ^b^	**4.15 ± 1.75 ^b^**	**11.01 ± 5.36 ^a^**
**A5**	1-Nonanal	1	Fatty, rose, citrus	tr	0.74 ± 0.00 ^b^	tr	tr	tr	0.58 ± 0.00 ^c^	**1.17 ± 0.00 ^a^**
**A7**	*(E,E)*-2,4-Heptadienal	10	Nutty, fatty	tr	0.89 ± 0.39	0.27 ± 0.04	tr	0.27 ± 0.11	0.89 ± 0.35	**1.11 ± 0.69**
**A8**	*(E,Z)*-2,6-Nonadienal	0.02	Green grass, cucumber	**9.54 ± 1.04 ^abc^**	**6.48 ± 0.00 ^cd^**	**7.85 ± 0.28 ^bcd^**	**9.74 ± 3.00 ^ab^**	**7.62 ± 0.95 ^bcd^**	**11.41 ± 0.02 ^a^**	**6.35 ± 1.09 ^d^**
**A9**	Benzeneacetaldehyde	4	Floral, rose; cherry-like	**3.96 ± 0.80 ^b^**	**7.62 ± 1.88 ^a^**	**4.92 ± 0.93 ^b^**	**7.94 ± 0.21 ^a^**	**7.64 ± 1.56 ^a^**	**3.03 ± 0.23 ^b^**	**8.35 ± 0.04 ^a^**
**Lactones**										
**L2**	*γ*-Octanoic lactone	6.5	Sweetness; coconut; creamy	0.76 ± 0.09 ^bc^	0.74 ± 0.23 ^bc^	0.95 ± 0.03 ^b^	0.53 ± 0.00 ^c^	0.76 ± 0.12 ^bc^	**1.26 ± 0.05 ^a^**	0.58 ± 0.07 ^c^
**L4**	*γ*-Decalactone	1.1	Peach-like, coconut, sweetness	tr	tr	**2.78 ± 0.00 ^a^**	tr	tr	**1.91 ± 0.00 ^b^**	tr
**Esters**										
**E1**	Ethyl acetate	5	Fruity, sweetness	**147.47 ± 28.34 ^d^**	**536.93 ± 44.40 ^a^**	**194.70 ± 149.42 ^cd^**	**341.03 ± 25.64 ^bc^**	**466.94 ± 29.25 ^ab^**	**323.05 ± 29.53 ^bcd^**	**291.44 ± 118.59 ^bcd^**
**Terpenoids**										
**T3**	Trans-rose oxide	5	Floral, rose	tr	tr	0.53 ± 0.02 c	tr	tr	**2.66 ± 0.00 ^a^**	**1.04 ± 0.00 ^b^**
**Volatile phenols**										
**P4**	4-Hydroxy-3-methoxystyrene	3	Caramel	**3.65 ± 0.19 ^b^**	**23.23 ± 12.57 ^a^**	**2.99 ± 0.14 ^b^**	**8.92 ± 0.57 ^b^**	**6.83 ± 1.86 ^b^**	**11.25 ± 0.75 ^b^**	**4.97 ± 0.42 ^b^**
**P7**	Eugenol	6	Lilac, honey, spice	tr	**12.14 ± 0.31**	tr	tr	**11.91 ± 0.11**	**12.14 ± 0.07**	tr
**P9**	3-Ethylphenol	1.7	Smoky, sweetness	**1.09 ± 0.00 ^c^**	**1.21 ± 0.00 ^a^**	tr	tr	tr	**1.20 ± 0.00 ^b^**	tr
**P10**	4-Hydroxystyrene	10	Almond	**8.73 ± 0.09**	**16.35 ± 5.22**	**8.66 ± 0.06**	**11.56 ± 0.35**	**11.27 ± 1.29**	**13.23 ± 0.71**	**10.09 ± 1.00**
**P11**	Guaiacol	3	Smoky	tr	**4.32 ± 1.34**	tr	**2.40 ± 0.29**	**2.36 ± 0.49**	**2.91 ± 0.16**	**2.31 ± 0.31**
**Alcohol**										
**O5**	cis-6-Nonen-1-ol	1	Green grass, cucumber	0.96 ± 0.07 ^e^	**84.68 ± 7.65 ^a^**	**1.4817 ± 0.4184 ^e^**	**23.59 ± 0.20 ^d^**	**44.44 ± 4.39 ^c^**	**72.87 ± 6.14 ^b^**	**70.20 ± 5.41 ^b^**

^1^ OAVs were calculated by dividing the concentrations by the odor thresholds. ^2^ Odor thresholds and Odor quality reference: [26,39,40,41,42,43,44] & http://foodflavorlab.cn/ (accessed on 20 June 2023); ^3^ bolded font indicates that the compound has an OAV > 1; data are presented as mean ± standard deviation of replicate trials. Different letters indicate a significant difference (*p* < 0.05); ^4^ “tr” indicates “trace”.

## Data Availability

Data are contained within the article or Supplementary Materials.

## Supplementary Materials

The following supporting information can be downloaded at: https://www.mdpi.com/article/10.3390/plants13020173/s1, Table S1: The information of the seven goji berry pulps; Table S2: The information of authentic flavor standards for qualitative and quantitation of aroma-active compounds Table S3: The load factor of sensory descriptors in each principal component; Table S4: Volatile aroma substance content in different goji berry pulps. Table S5 Standard regression coefficients of the volatile compounds to the aroma attribute variables [26,39,41,42,43,44,61,62].
