# Supplementary material for: Sensory-Guided Establishment of Sensory Lexicon and Investigation of Key Flavor Components for Goji Berry Pulp
Source: Plants (Basel). 2024 Jan 8;13(2):173. doi: 10.3390/plants13020173 (PMC10820852; doi:10.3390/plants13020173)
Supplement: Supplementary file 1 [file plants-13-00173-s001.zip › plants-2772153-supplementary.pdf]

Table S1. The information of the seven goji berry pulp.

Brand	Sample codes	Ingredients	Origin of raw materials	Manufacturers
FU DONG HAI	FDH	Goji berry juice	Ningxia	Guangdong Fengchun Pharmaceutical Co.
QING YUAN TANG	QYT	Fresh wolfberry, Citric acid	Ningxia	Ningxia Fushou Kangning Health Biotechnology Co.
NINGANPU	NAB	Fresh wolfberry, Vitamin C, Citric acid	Ningxia	Ningxia Ninganbao Local Specialties Co.
RUNDEMANOR	RDZY	Fresh wolfberry, Vitamin C, Citric acid	Ningxia	Ningxia Runde Biotechnology Co.
COFCO	ZL	Fresh wolfberry, Vitamin C, Citric acid, Water	Qinghai	Sirio Pharma Co., Ltd
QIZITOWN	QZT	/	Ningxia	Qizitown (Ningxia) Health Industry Co.
WOLFBERRY	WFBR	Goji berry juice	Ningxia	Ningxia Wolfram & Berry Wolfberry Industry Co.

Table S2. The information of authentic flavor standards for qualitative and quantitation of aroma-active compounds.

Flavor standards	CAS	Manufacturers	Purity
**Acids**			
Butyric Acid	107-92-6	Sigma-Aldrich	>99%
Hexanoic acid	142-62-1	Macklin	>99%
2-Ethylhexanoic acid	149-57-5	Aladdin	>99%
Octanoic acid	124-07-2	Aladdin	>99%
Decanoic acid	334-48-5	Aladdin	>99%
**Aldehydes**			
Hexanal	66-25-1	Macklin	>98%
Trans-2-Hexenal	6728-26-3	Aladdin	>98%
Octanal	124-13-0	Macklin	>99%

(E)-Hept-2-enal	18829-55-5	Aladdin	>99%
1-Nonanal	124-19-6	Macklin	>95%
(E)-2-Octenal	2548-87-0	Macklin	>95%
trans,trans-2,4-Heptadienal	4314-03-5	Macklin	>90%
2,6-Nonadienal, (E,Z)-	557-48-2	Aladdin	>95%
**Lactones**			
δ-Hexalactone	823-22-3	Aladdin	>99%
γ-Octanoic lactone	104-50-7	Macklin	>99%
5-Hydroxyoctanoic acid lactone	698-76-0	Macklin	>98%
γ-Decalactone	706-14-9	Aladdin	>98%
Undecanolactone	710-04-3	Aladdin	>98%
δ-Dodecalactone	713-95-1	Sigma-Aldrich	>98%
**Esters**			
Ethyl acetate	141-78-6	Macklin	>99%
Amyl acetate	628-63-7	DrE	>99%
Isoamyl acetate	123-92-2	Macklin	>99%
Methyl hexanoate	106-70-7	Macklin	>99%
Ethyl lactate	97-64-3	Macklin	>99%
Caprylic acid methyl ester	111-11-5	Adamas	>99%
Heptyl acetate	112-06-1	TCI	>98%
Ethyl 3-hydroxybutyrate	5405-41-4	Macklin	>99%
2-hydroxy4-methyl ethyl valerate	10348-47-4	Aladdin	>98%
Methyl salicylate	119-36-8	Macklin	>99%
Palmitic acid ethyl ester	628-97-7	Adamas	>99%
**Ketone**			
1-Octen-3-one	4312-99-6	Macklin	>96%

2-Nonanone	821-55-6	Macklin	>99%
**Terpenoids**			
(+)-Dipentene	5989-27-5	TCI	>99%
Terpinolene	586-62-9	TCI	>90%
4-Methyl-2-(2-methyl-1-propenyl)tetrahydropyran (cis-and trans-mixture)	16409-43-1	Sigma-Aldrich	>99%
Linalool	78-70-6	Macklin	>98%
Citronellyl acetate	150-84-5	Aladdin	>95%
Citronellol	106-22-9	Shanghai yuanye Bio-Technology Co., Ltd	>98%
alpha-Terpineol	98-55-5	Aladdin	>95%
Citral	5392-40-5	Macklin	>98%
Nerol	106-25-2	Adamas	>97%
Geranic acid	459-80-3	alfa aesar	>98%
Farnesol	4602-84-0	alfa aesar	>95%
**Volatile phenols**			
2-Methoxy-4-methylphenol	93-51-6	Aladdin	>98%
o-Cresol	95-48-7	Aladdin	>99%
4-Ethyl-2-methoxyphenol	2785-89-9	Aladdin	>98%
4-Hydroxy-3-methoxystyrene	7786-61-0	Sigma-Aldrich	>98%
p-Cresol	106-44-5	Macklin	>99%
m-Cresol	108-39-4	Aladdin	>99%
Eugenol	97-53-0	Macklin	>98%
4-Ethylphenol	123-07-9	CNW	>99%
3-Ethylphenol	620-17-7	DrE	>98%
4-Hydroxystyrene	2628-17-3	Jkchemical	>99%
Guaiacol	90-05-1	TCI	>99%

**Furan**			
Furfural	35796	Sigma-Aldrich	>99%
2-Acetylfuran	1192-62-7	Sigma-Aldrich	>99%
5-Methyl furfural	620-02-0	Macklin	>99%
Furfuryl alcohol	98-00-0	Adamas	>98%
**Alcohol**			
2-Methyl-1-propanol	78-83-1	Aladdin	>99%
1-Pentanol	71-41-0	Macklin	>99%
3-Methyl-1-butanol	123-51-3	Aladdin	>99%
2-Heptanol	543-49-7	Aladdin	>98%
cis-6-Nonen-1-ol	35854-86-5	Aladdin	>95%
1-Octen-3-ol	3391-86-4	Sigma-Aldrich	>99%
2,3-Butanediol	513-85-9	Macklin	>98%
1-Octanol	111-87-5	Macklin	>99%
Pyrazines			
2,3-Dimethyl-5-ethylpyrazine	15707-34-3	Sigma-Aldrich	>98%
Acetylpyrazine	22047-25-2	Sigma-Aldrich	>97%
**Benzene ring**			
o-Xylene	95-47-6	Macklin	>99%
Styrene	100-42-5	Macklin	>99%
Naphthalene	91-20-3	Macklin	>99%
**C13- norisoprenoid**			
Irisone	14901-07-6	Aladdin	>97%

Table S3. The load factor of sensory descriptors in each principal component

Sensory attributes	F1	F2	F3	F4	F5
**Aroma**					
Goji berry	0.520	0.293	-0.386	0.359	-0.522
Tomato	0.554	0.702	-0.365	0.117	0.139
Roast sweet potatoes	-0.039	-0.343	-0.080	0.716	0.601
Pumpkin	0.577	-0.772	0.236	0.100	0.020
Hay	0.486	0.375	-0.360	-0.227	0.665
Sweet	-0.580	-0.255	-0.719	0.203	-0.100
Acetic acid	-0.811	0.350	0.264	0.310	0.092
Honey	0.616	0.295	0.127	0.688	0.083
Jujube	-0.834	-0.387	0.036	-0.113	0.263
**Flavor / mouthfeel**					
Acidity	0.665	-0.709	-0.209	-0.079	0.059
Sweetness	-0.416	0.803	0.142	-0.062	0.237
Bitter	-0.068	-0.436	-0.521	-0.693	0.097
Astringency	0.281	-0.757	0.417	0.376	0.021
Goji berry	0.628	0.688	0.291	-0.162	-0.148
Tomato	0.913	-0.258	0.184	-0.201	-0.043
Granularity	-0.431	-0.016	0.863	-0.262	-0.011
Viscosity	-0.821	-0.154	-0.309	0.332	-0.311

a: Values with eigenvectors greater than 0.500 are in red; values with eigenvectors less than -0.500 are in blue.

Table S4. Volatile aroma substance content in different goji berry pulps.

No.	Compounds	Odor quality[a]	FDH	NAB	QYT	QZT	RDZY	WFBR	ZL
**Acids**									
**AC1**	Butyric acid	Acid	155.98 ± 14.23 a[b]	8.26 ± 1.33 d	87.18 ± 7.70 b	19.48 ± 2.84 d	14.55 ± 1.78 d	43.50 ± 6.54 c	13.17 ± 4.28 d
**AC2**	Hexanoic acid	Cheese, fatty	88.41 ± 5.95 c	678.38 ± 156.25 a	129.64 ± 4.13 c	259.31 ± 43.12 bc	339.24 ± 91.84 b	254.62 ± 31.18 bc	329.49 ± 77.77 b
**AC3**	2-Ethylhexanoic acid	Cheese	Tr[c]	1.83 ± 0.45 b	1.48 ± 0.40 b	1.30 ± 0.02 b	17.53 ± 6.29 a	2.24 ± 0.07 b	10.82 ± 2.88 a
**AC4**	Octanoic Acid	Cheese, fatty	5.04 ± 0.16	28.29 ± 14.16	6.11 ± 0.50	14.10 ± 0.69	19.95 ± 6.38	20.34 ± 1.65	15.72 ± 5.45
**AC5**	Decanoic acid	Acid, fatty	1.71 ± 0.12	4.54 ± 3.29	1.65 ± 0.04	1.34 ± 0.08	4.05 ± 0.97	4.30 ± 0.29	2.21 ± 1.17
**AC6**	Benzoic acid	Fatty	22.46 ± 7.95 b	54.74 ± 31.54 b	55.60 ± 1.10 b	46.72 ± 1.38 b	41.37 ± 4.23 b	99.53 ± 6.61 a	41.78 ± 20.89 b
**Aldehyde**									
**A1**	Hexanal	Apple-like, green grass, citrus	3.87 ± 0.32 c	20.08 ± 0.27 b	3.69 ± 1.13 c	45.94 ± 7.64 a	6.21 ± 0.93 c	9.36 ± 0.12 c	8.90 ± 0.82 c
**A2**	(*E*)-2-Hexenal	Green grass, banana-like	0.23 ± 0.00 d	5.32 ± 1.07 b	0.36 ± 0.04 d	2.58 ± 0.25 c	2.73 ± 0.12 c	2.51 ± 0.07 c	7.94 ± 0.29 a
**A3**	Octanal	Fruity, green grass, lemon	0.66 ± 0.64 b	0.84 ± 0.47 b	0.80 ± 0.29 b	tr	0.24 ± 0.03 b	2.91 ± 1.23 b	7.71 ± 3.76 a
**A4**	(*E*)-2-Heptenal	Green grass, fatty	tr	1.62 ± 0.32	tr	tr	0.25 ± 0.01	0.98 ± 0.57	2.67 ± 2.62
**A5**	Nonanal	Fatty, rose, citrus	tr	0.75 ± 0.00 b	tr	tr	tr	0.59 ± 0.00 c	1.17 ± 0.00 a
**A6**	(*E*)-2-Octenal	Green grass, nutty	tr	0.78 ± 0.21	tr	tr	0.24 ± 0.09	0.68 ± 0.24	0.83 ± 0.64
**A7**	(*E*,*E*)-2, 4-heptadienal	Nutty, fatty	tr	8.99 ± 3.93	2.73 ± 0.40	tr	2.77 ± 1.11	8.97 ± 3.55	11.12 ± 6.93
**A8**	(*E*,*Z*)-2, 6-Nonadienal	Green grass, cucumber	0.19 ± 0.02 abc	0.13 ± 0.00 cd	0.16 ± 0.01 bcd	0.20 ± 0.06 ab	0.15 ± 0.02 bcd	0.23 ± 0.00 a	0.13 ± 0.02 d
**A9**	Benzeneacetaldehyde	Floral, rose，	15.87 ± 3.22 b	30.51 ± 7.54 a	19.69 ± 3.76 b	31.80 ± 0.85 a	30.57 ± 6.27 a	12.13 ± 0.95 b	33.44 ± 0.19 a

		cherry							
**A10**	2*E*,4*E*-decadienal	/	tr	0.15 ± 0.07	tr	tr	0.08 ± 0.00	0.18 ± 0.05	0.14 ± 0.00
**Lactones**									
**L1**	δ-Hexalactone	Creamy，fruity，coconut	15.12 ± 1.28 cd	15.96 ± 5.76 cd	22.90 ± 1.96 bc	8.06 ± 0.73 d	21.86 ± 2.80 bc	36.72 ± 6.54 a	28.46 ± 9.66 ab
**L2**	γ-Octalactone	Sweet, coconut, creamy	5.00 ± 0.63 bc	4.81 ± 1.53 bc	6.21 ± 0.22 b	3.49 ± 0.00 c	5.00 ± 0.79 bc	8.23 ± 0.35 a	3.83 ± 0.48 c
**L3**	σ-Octalactone	Sweet, coconut creamy	1.67 ± 0.00 b	19.12 ± 10.14 a	1.62 ± 0.17 b	4.99 ± 0.55 b	9.53 ± 2.07 ab	9.08 ± 0.73 b	8.72 ± 2.75 b
**L4**	γ-Decalactone	Peach-like, coconut, sweet	tr	tr	3.06 ± 0.00 a	tr	tr	2.10 ± 0.00 b	tr
**L5**	δ-Undecalactone	Creamy, fatty, coconut, fruity, peach-like	0.79 ± 0.00	2.40 ± 0.00	tr	0.88 ± 0.02	0.56 ± 0.14	1.88 ± 1.46	0.53 ± 0.22
**L6**	δ-Dodecalactone	Peach-like, sweet	0.70 ± 0.02 c	1.89 ± 0.00 a	tr	0.56 ± 0.00 c	1.16 ± 0.00 b	0.78 ± 0.24 c	0.76 ± 0.10 c
**Esters**									
**E1**	Ethyl acetate	Fruity, sweet	737.39 ± 141.72 d	2684.66 ± 222.04 a	973.50 ± 747.14 cd	1705.18 ± 128.22 bc	2334.74 ± 146.27 ab	1615.29 ± 147.69 bcd	1457.25 ± 592.98 bcd
**E2**	Pentyl acetate	Fruity, banana	2.69 ± 1.02 c	1.05 ± 0.00 d	1.23 ± 0.00 d	2.65 ± 0.19 c	5.39 ± 0.00 b	25.37 ± 0.66 a	0.84 ± 0.00 d
**E3**	Isoamyl Acetate	Sweet, fruity	tr	0.41 ± 0.09 b	tr	tr	tr	0.79 ± 0.10 a	0.34 ± 0.00 b
**E4**	Methyl hexanoate	Fruity, sweet	2.23 ± 0.96 e	14.46 ± 0.73 bc	2.47 ± 2.33 e	20.11 ± 3.15 ab	25.12 ± 1.59 a	7.08 ± 5.32 de	10.02 ± 1.41 cd
**E5**	Ethyl lactate	Fruity,	1.28 ± 0.00 c	2.01 ± 0.00 bc	3.94 ± 2.00 b	tr	2.09 ± 0.00 bc	6.75 ± 0.47 a	1.84 ± 0.61 bc

		creamy							
**E6**	Methyl octanoate	Fruity, citrus	0.06 ± 0.00 c	0.58 ± 0.13 b	tr	0.42 ± 0.00 b	0.85 ± 0.16 a	0.17 ± 0.08 c	1.05 ± 0.08 a
**E7**	Acetic acid, heptyl ester	Fresh, fruity, apricot	0.06 ± 0.03 c	1.28 ± 0.16 a	0.09 ± 0.06 c	0.72 ± 0.06 ab	0.56 ± 0.01 bc	0.66 ± 0.16 bc	1.06 ± 0.63 ab
**E8**	Butanoic acid, 3-hydroxy-,ethyl ester	Grape, fruity, roast	tr	17.23 ± 8.90	0.54 ± 0.00	tr	tr	5.28 ± 1.61	24.15 ± 9.37
**E9**	Ethyl DL-2-Hydroxy-4-methylvalerate	Fresh, black berry	87.99 ± 10.77 ab	76.62 ± 6.69 bc	27.07 ± 3.38 d	74.13 ± 0.64 c	95.69 ± 3.82 a	83.99 ± 6.21 abc	24.99 ± 3.38 d
**E10**	Methyl salicylate	Green grass	0.86 ± 0.08 c	2.49 ± 0.95 ab	0.67 ± 0.18 c	1.29 ± 0.15 bc	2.65 ± 0.88 a	0.85 ± 0.23 c	0.64 ± 0.03 c
**E11**	Ethyl phenylacetate	Sweet, floral, honey, rose	0.04 ± 0.01 ab	tr	0.05 ± 0.01 a	tr c	0.03 ± 0.00 ab	0.02 ± 0.02 bc	tr
**E12**	Ethyl palmitate	Fruity, creamy	0.47 ± 0.00 b	0.22 ± 0.00 b	tr	tr	tr	1.27 ± 0.25 a	0.51 ± 0.00 b
**Ketones**									
**K1**	1-Octen-3-one	Mushroom, earthy	tr	0.59 ± 0.13 b	tr	tr	tr	0.41 ± 0.09 b	1.45 ± 0.00 a
**K2**	6-Methyl-5-heptene-2-one	Apple-like, banana, mung bean	0.46 ± 0.13 c	9.06 ± 0.67 a	0.72 ± 0.35 c	3.02 ± 0.30 bc	2.76 ± 0.26 bc	5.75 ± 1.23 ab	6.49 ± 4.07 ab
**K3**	2-Nonanone	Cheese, green grass, fruity, earthy	0.14 ± 0.00 e	1.57 ± 0.26 c	0.10 ± 0.00 e	1.10 ± 0.05 d	2.35 ± 0.34 b	0.42 ± 0.23 e	2.93 ± 0.20 a
**Terpenoids**									
**T1**	D-Limonene	Green grass, Apple-like, citrus	0.18 ± 0.04	0.81 ± 0.19	tr	0.71 ± 0.54	0.78 ± 0.21	0.24 ± 0.04	0.38 ± 0.18
**T2**	Terpinolene	Fresh, woody, sweet, citrus	tr	0.15 ± 0.04 b	tr	0.06 ± 0.00 b	0.40 ± 0.12 a	0.13 ± 0.00 b	0.17 ± 0.03 b
**T3**	trans-Rose oxide	Floral, rose	tr	tr	2.68 ± 0.15 c	tr	tr	13.32 ± 0.00 a	5.22 ± 0.00 b

**T4**	Citronellal	Lemon, green grass	tr	0.24 ± 0.06 b	0.07 ± 0.00 c	0.07 ± 0.00 c	0.12 ± 0.00 c	0.29 ± 0.04 b	0.43 ± 0.01 a
**T5**	Linalool	Citrus, floral, sweet, rose	0.43 ± 0.10 b	1.14 ± 0.28 a	0.39 ± 0.13 b	0.57 ± 0.01 b	1.29 ± 0.29 a	1.42 ± 0.34 a	1.24 ± 0.12 a
**T6**	Citronellyl acetate	Floral, green grass, rose, fruity	tr	0.41 ± 0.12	tr	tr	0.26 ± 0.00	0.41 ± 0.07	0.35 ± 0.09
**T7**	Citronellol	Citrus, green grass, sweet	tr	0.08 ± 0.00 a	tr	tr	0.07 ± 0.00 c	0.07 ± 0.00 b	tr
**T8**	α-Terpineol	Rosin, clove, floral, citrus	tr	tr	tr	tr	0.48 ± 0.39	0.48 ± 0.04	0.22 ± 0.00
**T9**	Citral	Lemon, sweet	tr	0.12 ± 0.05	tr	tr	0.04 ± 0.00	0.07 ± 0.01	0.06 ± 0.04
**T10**	Nerol	Sweet, citrus	0.68 ± 0.19 d	13.72 ± 0.09 a	1.16 ± 0.15 d	4.92 ± 0.32 c	6.94 ± 1.93 bc	9.61 ± 1.34 b	5.85 ± 3.00 c
**T11**	Geranic acid	Green	2.19 ± 0.17 b	6.68 ± 2.90 a	2.30 ± 0.01 b	5.75 ± 0.19 a	2.38 ± 0.29 b	2.28 ± 0.05 b	2.28 ± 0.23 b
**T12**	Farnesol	Sweet , floral	0.20 ± 0.00	1.00 ± 0.60	tr	0.31 ± 0.02	0.35 ± 0.10	0.55 ± 0.06	0.30 ± 0.14
**Volatile phenols**									
**P1**	4-Methylguaiacol	Clove, woody, smoky	tr	7.70 ± 0.00 a	tr	tr	tr	6.22 ± 0.00 b	tr
**P2**	o-Cresol	Chinese traditional medicine, leather, smoky	tr	2.15 ± 0.00 a	tr	1.66 ± 0.00 b	tr	2.53 ± 0.21 a	tr
**P3**	Phenol, 4-ethyl-2-methoxy-	Smoky, clove	tr	6.71 ± 0.00 b	tr	tr	6.54 ± 0.00 b	9.32 ± 0.55 a	6.47 ± 0.00 b
**P4**	4-Vinylguaiacol	Caramel	10.97 ± 0.60 b	69.70 ± 37.73 a	8.98 ± 0.43 b	26.78 ± 1.71 b	20.51 ± 5.60 b	33.77 ± 2.25 b	14.93 ± 1.27 b
**P5**	p-Cresol	Green grass,flora, animal	5.42 ± 0.01 c	5.92 ± 0.40 ab	5.56 ± 0.01 bc	5.67 ± 0.11 bc	5.53 ± 0.06 bc	6.16 ± 0.10 a	5.45 ± 0.12 c
**P6**	m-Cresol	Medicinal	6.38 ± 0.93	5.94 ± 0.31	6.56 ± 0.38	5.64 ± 0.27	5.69 ± 0.17	8.50 ± 1.92	5.85 ± 0.62

		incense, woody							
**P7**	Eugenol	Clove, honey , spice	tr	72.90 ± 1.87	tr	tr	71.48 ± 0.66	72.84 ± 0.43	tr
**P8**	4-Ethylphenol	Green grass, cheese	1.89 ± 0.08	2.34 ± 0.38	2.24 ± 0.10	tr	2.55 ± 0.30	2.91 ± 0.18	3.38 ± 0.72
**P9**	3-ethylphenol	Smoky, sweet	1.85 ± 0.00 c	2.07 ± 0.00 a	tr	tr	tr	2.06 ± 0.00 b	tr
**P10**	4-Vinylphenol	Almond	87.33 ± 0.94	163.59 ± 52.27	86.63 ± 0.63	115.70 ± 3.57	112.73 ± 12.91	132.35 ± 7.11	100.99 ± 10.06
**P11**	Guaiacol	Smoky	tr	13.11 ± 4.04	tr	7.23 ± 0.89	7.10 ± 1.49	8.74 ± 0.50	6.95 ± 0.95
**Furans**									
**F1**	Furfural	Sweet, almond	1949.19 ± 416.96 a	443.61 ± 145.47 cd	1229.66 ± 212.94 b	191.50 ± 9.92 d	802.83 ± 179.80 bc	653.75 ± 47.95 cd	867.60 ± 13.43 bc
**F2**	Acetylfuran	Sweet, nutty	55.31 ± 10.45 b	35.68 ± 9.79 bc	34.76 ± 3.00 bc	21.07 ± 1.94 c	78.66 ± 20.61 a	25.18 ± 1.48 c	79.62 ± 5.78 a
**F3**	5-MethyIfurfural	Creamy	15.31 ± 3.23 b	4.46 ± 1.25 d	14.40 ± 0.53 bc	1.61 ± 0.27 d	13.35 ± 3.69 bc	9.78 ± 0.06 c	21.42 ± 1.97 a
**F4**	Furfuryl alcohol	Alcohol , chemical, sweet, creamy	255.41 ± 11.04	212.18 ± 66.11	209.36 ± 12.08	247.72 ± 17.44	355.23 ± 20.99	243.50 ± 34.47	403.10 ± 119.51
**Alcohol**									
**O1**	Isobutanol	Alcohol	0.61 ± 0.24	tr	0.22 ± 0.07	tr	0.52 ± 0.00	0.85 ± 0.83	0.80 ± 0.07
**O2**	1-Pentanol	Sweet, oil	5.92 ± 0.71 d	23.19 ± 1.72 ab	7.30 ± 1.60 cd	24.48 ± 0.65 a	12.26 ± 1.35 bcd	18.25 ± 0.96 abc	24.95 ± 13.07 a
**O3**	Isopentanol	Fruity, banana	20.77 ± 4.02	19.99 ± 2.22	25.11 ± 14.27	11.63 ± 0.61	23.52 ± 0.04	30.25 ± 9.93	18.37 ± 3.41
**O4**	2-Heptanol	Fruity, plant-like	0.12 ± 0.02 b	tr	0.17 ± 0.00 a	tr	tr	0.17 ± 0.00 a	0.12 ± 0.01 b
**O5**	(Z)-6-Nonenol	Green grass, cucumber	0.97 ± 0.07 e	84.68 ± 7.65 a	1.48 ± 0.42 e	23.59 ± 0.20 d	44.45 ± 4.39 c	72.87 ± 6.15 b	70.20 ± 5.41 b
**O6**	1-Octen-3-ol	Mushroom	0.35 ± 0.00 d	3.36 ± 0.49 a	0.47 ± 0.00 cd	1.86 ± 0.18 bc	1.61 ± 0.16 bcd	1.73 ± 0.34 bcd	2.76 ± 1.48 ab

**O7**	2, 3-Butanediol	Creamy, buttery	7.42 ± 3.10 bc	6.64 ± 1.53 c	6.53 ± 1.22 c	5.37 ± 0.00 c	7.92 ± 0.83 bc	11.60 ± 2.59 ab	15.15 ± 2.47 a
**O8**	1-octanol	Woody, green grass, blueberry	0.21 ± 0.06	0.65 ± 0.25	0.31 ± 0.08	0.46 ± 0.02	0.80 ± 0.31	0.87 ± 0.05	0.82 ± 0.30
**O9**	2-Phenylethanol	Citrus, rose	75.09 ± 3.52 bc	73.19 ± 15.21 bc	102.18 ± 6.95 a	70.69 ± 7.41 bc	76.13 ± 9.03 bc	81.76 ± 2.82 b	58.83 ± 7.15 c
**Pyrazines**									
**PY1**	5-Ethyl-2, 3-dimethylpyrazine	Caramel, roast	0.65 ± 0.12 a	tr	0.74 ± 0.01 a	tr	0.05 ± 0.07 c	0.27 ± 0.04 b	tr
**PY2**	Acetylpyrazine	Roast	1.02 ± 0.28 ab	0.48 ± 0.30 c	1.31 ± 0.04 a	0.31 ± 0.04 c	0.24 ± 0.01 c	0.39 ± 0.14 c	0.61 ± 0.26 bc
**Benzenes**									
**B1**	o-Xylene	Sweet, floral	21.25 ± 8.46	19.57 ± 2.61	1.20 ± 1.14	27.51 ± 10.98	25.29 ± 18.19	45.00 ± 36.16	5.98 ± 1.72
**B2**	Styrene	Floral	tr	0.08 ± 0.03	tr	0.25 ± 0.11	0.25 ± 0.07	tr	0.30 ± 0.00
**B3**	p-Cymene	Citrus, lemon, fruity	tr	tr	tr	tr	0.04 ± 0.00 a	0.05 ± 0.00 a	0.03 ± 0.00 b
**B4**	o-Cymene	/	0.05 ± 0.00 c	0.10 ± 0.05 c	tr	0.09 ± 0.04 c	0.34 ± 0.06 a	0.24 ± 0.02 b	0.05 ± 0.00 c
**B5**	Naphthalene	Hay	tr	0.18 ± 0.00	tr	tr	0.15 ± 0.04	0.11 ± 0.01	tr
**B6**	2-phenoxyethanol	Rose, spice	38.83 ± 0.33 b	38.54 ± 1.06 b	41.02 ± 0.14 b	39.40 ± 0.52 b	41.56 ± 1.31 b	118.63 ± 12.35 a	39.74 ± 1.00 b
**C_{13}-norisoprenoids**									
**C1**	Damascenone	Sweet, fruity	0.11 ± 0.00 b	0.27 ± 0.11 a	0.10 ± 0.00 b	0.13 ± 0.00 b	0.10 ± 0.02 b	0.09 ± 0.00 b	0.18 ± 0.02 ab
**C2**	*β*-Ionone	Rose, violets	0.35 ± 0.06 d	1.73 ± 0.53 a	0.30 ± 0.02 d	1.41 ± 0.08 ab	0.99 ± 0.30 bc	1.13 ± 0.05 bc	0.66 ± 0.22 cd

[a] Odor quality references [26,39,41–44,61,62] & http://foodflavorlab.cn/;

[b] Data are expressed as mean ± standard deviation of repeated trials. Different letters indicate significant differences ($p<0.05$) ;

[c] "tr" indicate "trace".

Table S5. Standard regression coefficients of the volatile compounds to the aroma attribute variables.

Attributes	No.	Compounds	Standard coefficient [a]	VIP	Odor quality [d]
**Goji berry**	A8[b]	*(E,Z)*-2,6-Nonadienal	0.298	0.883	Green grass, cucumber
	B1	o-Xylene	0.236	1.090	Sweet, floral
	E9	2-hydroxy4-methyl ethyl valerate	0.180	1.111	Fresh, blackberry
	A1	Hexanal	0.117	1.193	Apple-like, green grass, citrus
	PY2[c]	Acetylpyrazine	-0.114	1.090	Roast
	L4	γ-Decalactone	-0.135	1.157	Peach-like, coconut, sweetness
	P8	4-Ethylphenol	-0.162	1.158	Green grass, cheese
	O9	Phenylethyl Alcohol	-0.167	1.039	Citrus, rose
**Tomato**	A8	*(E,Z)*-2,6-Nonadienal,	0.219	1.307	Green grass, cucumber
	B1	o-Xylene	0.184	1.351	Sweet, floral
	E12	Palmitic acid ethyl ester	0.176	1.542	Fruity, creamy
	E9	2-hydroxy4-methyl ethyl valerate	0.157	0.854	Fresh, blackberry
	P9	3-Ethylphenol	0.154	1.183	Smoky, sweet
	O1	2-Methyl-1-propanol	0.126	0.886	Alcohol

	AC6	Benzoic acid	-0.125	0.893	Fatty
	AC3	2-Ethylhexanoic acid	-0.147	0.801	Cheese
	E10	Methyl salicylate	-0.156	0.834	Green grass
	O9	Phenylethyl Alcohol	-0.299	0.891	Citrus, rose
**Roast sweet potatoes**	E11	Benzeneacetic acid, ethyl ester	0.176	1.093	Sweet, floral, honey, rose
	F1	Furfural	0.171	0.822	Sweet, almond
	E9	2-hydroxy4-methyl ethyl valerate	0.149	1.090	Fresh, blackberry
	E10	Methyl salicylate	0.100	0.806	Green grass
	A1	Hexanal	-0.154	1.198	Apple-like； green grass, citrus
	O2	1-Pentanol	-0.207	1.212	Sweet, oil
**Pumpkin**	AC3	2-Ethylhexanoic acid	0.202	1.064	Cheese
	T2	Terpinolene	0.166	1.215	Fresh, woody, sweet, citrus
	K3	2-Nonanone	0.130	1.075	Cheese, green grass, fruity, petrichor
	E6	Caprylic acid methyl ester	0.124	1.081	Fruity, citrus-like
**Hay**	A8	*(E,Z)*-2,6-Nonadienal	0.103	0.949	Green grass, cucumber
**Sweet**	F3	5-Methyl furfural	0.108	0.879	Creamy

	T11	Geranic acid	-0.118	1.068	Green
	P4	4-Hydroxy-3-methoxystyrene	-0.090	1.287	Smoky, sweet
	P2	o-Cresol	-0.094	1.106	Chinese traditional medicine, leather, smoky
**Honey**	E9	2-hydroxy4-methyl ethyl valerate	0.391	1.508	Fresh, blackberry
	B1	o-Xylene	0.266	1.240	Sweet, floral
	P9	3-Ethylphenol	0.255	1.117	Smoky, sweet
	L4	γ-Decalactone	-0.237	0.854	Peach-like, coconut, sweetness
**Acetic acid**	AC1	Butyric Acid	0.151	1.305	Acid
	AC2	Hexanoic acid	0.130	1.117	Cheese
	E4	Methyl hexanoate	-0.107	1.047	Fruity, sweet
	B4	o-Cymene	-0.121	0.936	/
	B2	Styrene	-0.136	0.988	Floral
	T10	Nerol	-0.158	0.954	Lilac, floral, citrus
	B3	*p*-Cymene	-0.163	0.974	Citrus, lemon, fruity
**Jujube**	O9	Phenylethyl Alcohol	0.456	1.268	Citrus, rose
	L4	γ-Decalactone	0.309	0.975	Peach-like, coconut, sweetness
	E11	Benzeneacetic acid, ethyl ester	0.152	1.151	Sweet, floral, honey,

				rose
PY2	Acetylpyrazine	0.100	0.953	Roast
L5	Undecanolactone	-0.288	0.946	Creamy, fatty, coconut, fruity, peach-like

[a] Only volatile aroma substances with absolute values of standard coefficients greater than 0.1 are shown

[b] Positive correlation of volatile aroma substances in red;

[c] Negative correlation of volatile aroma substances in blue;

[d] Description of aroma, to see Table 4.

References

26. Oğuz, İ.; Oğuz, H.I.; Kafkas, N.E. Evaluation of fruit characteristics of various organically-grown goji berry (*Lycium barbarum* L., *Lycium chinense* Miller) species during ripening stages. *J. Food Compos. Anal.* **2021**, *101*, 103846.
39. Kim, J.-S.; Chung, H.Y. GC-MS Analysis of the Volatile Components in Dried Boxthorn (*Lycium chinensis*) Fruit. *J. Korean Soc. Appl. Biol. Chem.* **2009**, *52*, 516–524. https://doi.org/10.3839/jksabc.2009.088.
41. Niu, Y.; Wang, R.; Xiao, Z.; Zhu, J.; Sun, X.; Wang, P. Characterization of ester odorants of apple juice by gas chromatography-olfactometry, quantitative measurements, odour threshold, aroma intensity and electronic nose. *Food Res. Int.* **2019**, *120*, 92–101. https://doi.org/10.1016/j.foodres.2019.01.064.
42. Tamura, H.; Ueno, S.; Naka, A.; Zhao, H.; Yonekura, L.; Isogai, T.; Wakui, R.; Shiota, M. Characterisation of aroma profile and evaluation of aroma quality in sweet cream butter. *Int. Dairy J.* **2021**, *114*, 104935. https://doi.org/10.1016/j.idairyj.2020.104935.
43. Tan, F.; Wang, P.; Zhan, P.; Tian, H. Characterization of key aroma compounds in flat peach juice based on gas chromatography-mass spectrometry-olfactometry (GC-MS-O), odor activity value (OAV), aroma recombination, and omission experiments. *Food Chem.* **2022**, *366*, 130604. https://doi.org/10.1016/j.foodchem.2021.130604.
44. Tang, W.M.; Chan, E.; Kwok, C.Y.; Lee, Y.K.; Wu, J.H.; Wan, C.W.; Chan, R.Y.; Yu, P.H.; Chan, S.W. A review of the anticancer and immunomodulatory effects of Lycium barbarum fruit. *Inflammopharmacology* **2012**, *20*, 307–314. https://doi.org/10.1007/s10787-011-0107-3.
61. Lee, G.H.; Shin, Y.; Oh, M.J. Aroma-active components of Lycii fructus (kukija). *J. Food Sci.* **2008**, *73*, C500–C505. https://doi:10.1111/j.1750-3841.2008.00851.x.
62. Li, X.M.; Li, X.L.; Zhou, A.G. Evaluation of antioxidant activity of the polysaccharides extracted from *Lycium barbarum* fruits in vitro. *Eur. Polym. J.* **2007**, *43*, 488–497. https://doi.org/10.1016/j.eurpolymj.2006.10.025.
